# The Importance of Sex Stratification in Autoimmune Disease Biomarker Research: A Systematic Review

**DOI:** 10.3389/fimmu.2018.01208

**Published:** 2018-06-04

**Authors:** Kristy Purnamawati, Jamie Ann-Hui Ong, Siddharth Deshpande, Warren Kok-Yong Tan, Nihar Masurkar, Jackson Kwee Low, Chester Lee Drum

**Affiliations:** ^1^Biomedical Institute for Global Health Research and Technology (BIGHEART), National University of Singapore (NUS), Singapore, Singapore; ^2^National University of Singapore, Singapore, Singapore; ^3^Cardiovascular Research Institute, National University Health System, Singapore, Singapore; ^4^Department of Medicine, Yong Loo Lin School of Medicine, National University of Singapore, Singapore, Singapore; ^5^Translational Laboratory in Genetic Medicine, Agency for Science, Technology and Research, Singapore, Singapore; ^6^Department of Surgery, Yong Loo Lin School of Medicine, National University of Singapore, Singapore, Singapore; ^7^Department of Biochemistry, Yong Loo Lin School of Medicine, National University of Singapore, Singapore, Singapore

**Keywords:** autoimmune diseases, sex differences, gender, sex stratification, biomarkers

## Abstract

The immune system is highly dynamic and regulated by many baseline characteristic factors. As such, significant variability may exist among different patient groups suffering from the same autoimmune disease (AD). However, contemporary research practices tend to take the reductionist aggregate approach: they do not segment AD patients before embarking on biomarker discovery. This approach has been productive: many novel AD biomarkers have recently been discovered. Yet, subsequent validation studies of these biomarkers tend to suffer from a lack of specificity, sensitivity, and reproducibility which hamper their translation for clinical use. To enhance reproducibility in validation studies, an optimal discovery-phase study design is paramount: one which takes into account different parameters affecting the immune system biology. In this systematic review, we highlight need for stratification in one such parameter, i.e., sex stratification. We will first explore sex differences in immune system biology and AD prevalence, followed by reported sex-bias in the clinical phenotypes of two ADs—one which more commonly affects females: systemic lupus erythematosus, and one which more commonly affects males: ankylosing spondylitis. The practice of sex stratification in biomarker research may not only advance the discovery of sex-specific AD biomarkers but more importantly, promote reproducibility in subsequent validation studies, thus easing the translation of these novel biomarkers from bench to bedside to improve AD diagnosis. In addition, such practice will also promote deeper understanding for differential AD pathophysiology in males and females, which will be useful for the development of more effective interventions for each sex type.

## Introduction

Autoimmune diseases (ADs) are a complex class of diseases resulting from the immune system failure to differentiate between self and foreign antigens (1). This misrecognition directs the immune system to attack self-antigens, which consequently modifies the biological functions of the affected tissues. Ultimately, tissue damage and dysfunctions ensue and present as clinical symptoms. However, the onset of clinical symptoms is often delayed and occurs following irreversible damage to the affected tissues or organ. There is a global urgency for the discovery of specific and sensitive biomarkers for an early detection of ADs. Additionally, the ideal AD biomarker(s) should also be surrogate for disease severity, progression to disability and response to therapy (2).

The discovery of such biomarkers is not straightforward. Currently, validated biomarkers do not yet fulfill this tall order (we list validated biomarkers from select ADs in Table S1 in Supplementary Material). The immune system biology is dynamic—it varies with genetic background, age, sex, and the environment (3–11). Thus, patients with varying characteristics may present with different clinical phenotype and biomarkers despite suffering from the same AD. Conventional biomarker research strategy has been reductionist and aggregated: they compare all AD patients and controls of mixed baseline characteristics. This approach, although suboptimal, has been productive, leading to the discovery of many novel AD biomarkers (2). Yet, validation and clinical translation of such novel biomarkers have proven to be challenging, possibly due to: (1) lack of control and patient group stratification and matching; (2) inappropriate biomarker validation strategy; and/or (3) techniques used in clinical trials (12, 13). In this review, we highlight the importance of sex stratification in AD biomarker research prior to the discovery-phase, by drawing attention to the fact that: (1) ADs display stronger female bias and (2) present with different disease trajectories in males and females. There are apparent sex differences in AD pathophysiology. These need to be recognized and hopefully over time, incorporated into AD research efforts, clinical diagnosis, and management for a better patient outcome.

## Methods

We assembled a comprehensive list of disease prevalence and associated biomarkers in females and males from different geographical locations. Extensive literature review for the study variables of interest for each of the diseases was mainly carried out using PubMed, although some books and online resources were also consulted. Standard search strategies were used, including medical subject headings such as “disease name” and “biomarker,” or “disease name” and other parameters of interest (e.g., “prevalence,” “in Japan,” “diagnosis,” “autoantibodies,” etc.). Of note, we searched in futility for “sex/gender differences,” “disease name,” and “biomarkers,” perhaps highlighting the paucity of such studies. In total, we reviewed >1,000 abstracts and >400 full papers and included papers which fulfilled the following criteria:

1. Table 1, review papers and an immunology textbook describing sex-biased immune responses in humans, i.e., Ref. (3, 4, 14).
2. Table 2, clinical studies indicating numbers of females and males in their study. Studies in which the male and female numbers were not representative of the disease prevalence and incidence in that particular geography were excluded.
3. Tables 3–6, only reviews [i.e., Ref. (15–19)] or primary clinical research papers reporting at least one significant (*P* < 0.05) sex-bias in clinical phenotype was reported.
4. Table S1 in Supplementary Material, review papers and primary research papers that propose novel serum- or plasma-derived proteomic biomarkers. Only biomarkers that have been validated in multiple clinical studies are included. Often this means seeing multiple papers reporting the same biomarkers. Exclusion criteria include:
  - generic biomarkers such as metabolites and oxidative stress biomarkers, likely to be observed with other diseases;
  - genetic biomarkers such as DNA, RNA, single nucleotide polymorphisms, etc.;
  - biomarkers derived from site-specific fluids, such as synovial and cerebrospinal fluids, urine, tears, fecal matter, etc.

**Table 1:** Sex dimorphism of the immune system biology.

Immune component	Cytokines (14)	Sex differences (females vs males) (3)	Effects of sex hormones (3, 4, 24)		
			
			Estradiol	Progesterone	Androgens
**Innate immunity**					
Toll-like receptor (TLR) pathway	Inflammatory cytokines, chemotactic factors, antimicrobial peptides, type I interferons (IFNα and IFNβ)	↑ TLR gene expression ↑ TLR7 expression ↓ IL-10 production by TLR-9 stimulated PBMCs	↑ TLR4, TLR7, TLR9	↓ TLR3, TLR7	↓ TLR4

Antigen-presenting cells (APCs)	Interleukins: IL 12, IL 17 (20)	↑ APC efficiency	↓ Antigen presentation (21)	↑ Antigen presentation (22)	↓ Antigen presentation (23)

Dendritic cells (DCs)	Interferons: IFNα Interleukins: IL10, IL12, IL23, IL27, IL28, IL29, IL37	↑ TLR7 activity ↑ Type I interferon (IFN) activity	↑ Activation, TLR7, TLR9, ↑ Production of CCL2, IL6, IL8 and IL12 ↑ Expansion of IFNγ-producing killer DCs from mature splenic DCs ↓ CXCL10, IFNα	↑ IL10, IL18, CD11c ↓ CD40, CD80, CD86	Not defined

Macrophage	Colony stimulating factors: GM-CSF Interferons: IFNα, Interleukins: IL1α, IL1β, IL6, IL10, IL12, IL15, IL18, IL23, IL27, oncostatin TNF family: TNFα	↑ Activation levels ↑ Phagocytic capacity ↑ IL-10 production ↓ Pro-inflammatory cytokine production ↓ TLR4 expression	↑ TLR4 ↓ IL1β, IL6 and TNF production	↑ FIZZ1, YM1 ↓ iNOS, NO	↓ TNF and iNOS/NO

Eosinophil	IFNα, IL16		↓ Count and mobilization	↑ Count	Not defined

Neutrophil	IFNγ, IL17	↑ Phagocytic capacity ↓ TLR expression levels	↑ Count, anti-inflammatory activity, elastase release ↓ Chemotactic activity	Not defined	↑ Count ↓ Kinases and Leukotriene formation

NK Cells	Interferons: IFNγ Interleukins: IL17, IL26, IL32 TNF family: TNFα	↓ Cell count	↑ IFNγ, Granzyme B ↓ FASL ↓ Cytotoxic activity	↑ Cell count and apoptosis (caspase dependent)	Not defined
**Adaptive immunity**					
T-cells	Colony stimulating factors: GM-CSF, M-CSF Interferons: IFNγ TNF family: TNFα, lymphotoxin, CD40L, FasL, CD27L, CD30L, APRIL, LIGHT, BAFF Interleukins: IL2, IL4, IL5, IL6, IL9, IL10, IL13, IL16, IL24, IL32, oncostatin T_H_1 cells: IL20 T_H_2 cells: IL21, IL25, IL31 T_H_17 cells: IL17, IL26 T_reg_: IL35	↑ CD4+ T cell count (25) ↑ CD4/CD8 T cell ratio ↑ Activated T cells count ↑ T cell proliferation ↓ CD8+ count ↑ CD8+ cytotoxicity ↑ T_H_2 bias ↓ T_H_1 bias ↓ T_reg_ cell count ↑ T_H_1 cytokine secretion	Low estradiol: ↑ IFNγ and T_H_1 cells responses High estradiol: ↑ IL4 and T_H_2 cells responses ↑ T_reg_ cell count ↑ Response of CD8+ T cells ↑ secretion of IFNγ and IL10 (24) ↑ CD4+ CCR1 and CCR5 expression ↓ T_H_17 cell count and IL17 expression ↓ CD4+ TNF production	↓ T_H_1 cells activity ↑ T_H_2 cells activity ↓ % of T_H_17 ↓ % of T_reg_ ↓ Response of CD8+ T cells	↓ IFNγ production by T_H_1 cells ↓ T_H_2 expression of IL4, IL5 and GATA3 ↑ IL17 ↑ T_reg_ cell count ↓ CD8+ cell count and activity

B-cells	Lymphotoxin	↑ B-cell count ↑ Antibody production	↑ IgM and IgG prodction ↑ Survival of autoreactive B cells ↓ Negative selection of naïve B cells	↑ Total antibody production ↓ Autoantibodies	↓ Response

**Table 2:** Female-to-male ratio of autoimmune diseases (ADs) from different regions of the world.

Class	Female: Male ratio				
	
Disease	North Americas	Scandinavia	Europe	Australia or New Zealand	Asia
**Systemic and connective tissue**					
Systemic lupus erythematosus	6 (26)–11.6 (27):1	4.7:1 (28) (Sweden)	5.8:1 (29) (France)	4.4:1 (30)	7.8 (31)–11.4 (32):1 (China) 6:1 (33) (Korea) 8.2:1 (34) (Japan)

Sjögren’s syndrome	5.5:1 (35)	8 (28)–8.7 (36):1 (Sweden)	15.8:1 (37) 18.6:1 (38) (Spain)	8.3:1 (39)	4.2:1 (40) (India) 17.4:1 (41) (Japan) 17:1 (42) (China)

**Pulmonary**					
Idiopathic pulmonary fibrosis	0.9 (43)–1.2 (44):1	0.3:1 (45) (Denmark) 0.4:1 (46) (Sweden)	0.7:1 (47) (Spain) 0.6:1 (48) (UK) 0.3:1 (49) (Germany)	0.5:1 (50)	0.3:1 (51) (Korea) 0.4:1 (52) (Japan)

**Skin**					
Scleroderma (also systemic)	4.8:1 (53)	3.2:1 (28) (Denmark) 3.8:1 (54) (Norway)	9.7:1 (55) (Italy) 4.7 (56)–7.2:1 (57) (UK) 10.4:1 (58) (France)	7.4:1 (59)	7.7:1 (34) (Japan) 4 (60) – 10 (61):1 (India)

Psoriasis	1 (62)–1.3 (63):1	1.1:1 (28) (Denmark) 1:1 (64) (Norway)	1.3:1 (65) (Spain) 0.8:1 (66) (Germany) 1:1 (67) (UK)	2:1 (68)	0.6:1 (69) (Japan)

**Hematopoetic and vascular**					
Antiphospholipid syndrome	3.6:1 (70, 71)	4.5:1 (72) (Norway)	2.1:1 (73) (Spain) 1.7:1 (74) (Italy) 10:1 (75) (UK)		5.4:1 (76) (Japan) 4.4:1 (77) (Singapore)

Immune thrombocytopenic purpura	1.1 (78)–1.4 (79, 80):1	1.7:1 (81) (Denmark)	1.7:1 (82) (France) 1.4:1 (83) (Germany) 1.3:1 (84) (UK)	1.3 (85)–1.6 (86, 87):1	1.6 (88)–2.2 (89):1 (China) 1.9:1 (90) (India) 4.3:1 (91) (Singapore)

**Endocrine**					
Grave’s disease	6:1 (92)	5.8:1 (93) (Denmark)	3.5:1 (94) (Greece) 4.4:1 (95) (France)	4:1 (96)	2.5–2.8:1 (97) (China)

Hashimoto’s thyroiditis	11.8:1 (98)	4.4:1 (28)	5.4:1 (94) (Greece)	7.3:1 (99)	10.7:1 (100) (China) 21.7:1 (101) (India)

Type 1 diabetes (adult*)	1:1 (102)	0.8:1 (103)	0.5:1 (104) (Italy) 0.6:1 (104) (Spain) 0.5–0.8:1 (104, 105) (UK) 1:1 (105) (Germany)	0.9–1.5:1 (105, 106)	1.3:1 (107) (India) 1.4:1 (108) (Japan)

**Gastrointestinal and liver**					
Primary biliary cholangitis	10 (109)–12.4 (110):1	4.1:1 (28)	7.9:1 (111) (France) 12.6:1 (112) (UK)	9:1 (113)	10.5:1 (114) (China) 6.5:1 (115) (Japan)

Autoimmune hepatitis	4.1:1 (116)	3.17:1 (117) (Sweden) 4:1 (118) (Norway) 2.6:1 (119) (Denmark)	5.5:1 (120) (Spain) 7.1:1 (121) (Italy) 2.7:1 (122) (Germany)	2.7:1 (123) (NZ) 3:1 (124) (AUS)	6.7:1 (125) (Japan) 5.9:1 (126) (China) 8.4:1 (127) (India) 11:1 (128) (Singapore)

Ulcerative colitis	0.9:1 (129)	1:1 (72)	0.9:1 (70) (France) 0.8:1 (71) (Western EU)	1.1:1 (71)	1.05:1 (76) (India) 0.7:1 (77) (Asia) 0.9:1 (73) (Japan)

Crohn’s disease	1:1 (129)	1.1:1 (28)	1.32:1 (70) (France) 0.7:1 (71) (Western EU)	1:1 (71)	1:1 (76) (India) 0.6:1 (77) (Asia) 0.4:1 (73) (Japan)

Celiac disease (adult^a^) (CoD)	1.3 (74)–2.7 (75):1	1.8:1 (28) (Denmark) 1.2:1 (130) (Finland) 2.4:1 (131) (Sweden)	1:1 (130) (Germany) 0.6:1 (130) (Italy) 0.5:1 (130) (UK)	1.6:1 (132)	0.7:1 (133) (India) 1.3:1 (134) (China)

**Musculoskeletal**					
Ankylosing spondylitis	0.3:1 (135)	0.5:1 (28) (Denmark) 0.4:1 (136) (Finland) 0.5:1 (137) (Sweden)	0.2:1 (138) (Greece)	0.4:1 (139)	0.3:1 (140) (China) 0.2:1 (141) (India) 0.2–0.3:1 (142) (Japan)

Rheumatoid arthritis	2.6:1 (143)	2.2:1 (28) (Denmark)	2.2:1 (144) (UK)		3.8:1 (140) (China)

Psoriatic arthritis	0.7:1 (145)	1.23:1 (137) (Sweden) 0.6:1 (146) (Norway)	0.2:1 (138) (Greece)		

**Neurological**					
Multiple sclerosis	2.6:1 (147)	2 (148)–2.3 (28):1 (Denmark)	2.4:1 (149) (France)	2.3 (150)–4.5 (151):1	1.8:1 (152) (China) 2.9:1 (153) (Japan) 1.7:1 (154) (India)

Myasthenia gravis	1.4:1 (155)	1.1:1 (28)	1.9:1 (156) (Italy) 1.4:1 (157) (France)	1.3:1 (158)	2:1 (159) (Japan) 0.4:1 (160) (India) 1.15:1 (161) (China)

Guillain–Barré syndrome	0.8:1 (162)	0.6:1 (163) (Finland) 0.8:1 (164) (Sweden)	0.6:1 (165) (Italy) 0.6 (166)–0.8 (167):1 (UK)	0.6:1 (168)	0.7:1 (169) (India) 0.7:1 (170) (China) 0.6:1 (171) (Japan)

*Data reflect non-pregnant females*.

*^a^Adult: 15 ≤ Age ≤ 65*.

**Table 3:** Sex differences in systemic lupus erythematosus clinical phenotypes.

Clinical phenotype	Studies showing phenotype is increased in males	Studies showing phenotype is increased in females	Studies showing statistically in insignificant in males and females
Mortality	(27, 172)		
Disease activity	(17, 27, 173)		(174)
Alopecia		(18, 27, 32, 175–182)	(183)
Photosensitivity		(18, 27, 176, 178, 179, 184, 185)	(181, 183)
Discoid lesions	(32, 181, 186–188)		(183)
Malar rash		(18, 27, 181, 186, 189)	(183)
Raynaud’s phenomenon (RP)		(17, 27, 32, 176–179, 184, 185, 190, 191)	
Musculoskeletal (myositis, tendonitis, arthralgia/arthritis)	(178)	(17, 18, 27, 173, 176, 177, 179, 185, 186, 188, 192, 193)	(16, 174, 181, 183)
Oral ulcers		(18, 27, 181, 184, 194)	(183)
Serositis	(17, 18, 175, 178, 181, 182, 184, 187)		(174, 183, 186)
Gastrointestinal complications	(179)		
Renal disease	(17, 18, 27, 174, 176, 177, 185, 187–191, 195, 196)		(16, 181, 183, 186)
Neurological and psychiatric disease	(182, 185), Seizure (197), peripheral neuropathy (17)	Psychosis (177), psychiatric (17, 175)	(181, 183, 186)
Hematological: thrombocytopenia, leukopenia	(18, 27, 177, 195)	(17, 174, 175, 182, 184, 188, 191)	(181, 183, 186)
Cardiovascular	(27, 176, 179, 187)		(181)
Thromboses	(27, 179, 180, 190, 193)		
Other	Constitutional symptoms: fever, weight loss (176), pleuritis (181), dry mouth and dry eyes (185)	Flares/severe flares (32); cutaneous (174); more frequent relapses (17), erythrocyte sedimentation rate, antinuclear antibody, anti-SSA, anti-SSB (181)	Mucocutaneous (16, 193), vasculitis (181, 186), low C3, anti-dsDNA, anti-Sm, anti-rRNP (181, 193)

*Adapted with modifications from Ref. (15–18)*.

*The results depict studies where significant (*P* < 0.05) differences were detected*.

**Table 4:** Comparative studies of male and female lupus: main clinical and demographic findings, adapted with modifications from Ref. (1, 8, 15–17).

**Year of Study**	**Country (ethnicity)**	**Study type**	**Size (%male)**	**Age at onset**	**Clinical phenotype (***P*** < 0.05)**		**Serology**
						
					**Increased in males**	**Increased in females**
**North America**							

NA	US (multiethnic) (173)	Prospective	618 (10.2)	37.1 (M), 36.5 (F)	Renal disease	Musculoskeletal	LAC (M)
1969–1983	US (198)	Inception	361 (17.2)	44.7 (M), 35.2 (F)	Seizures		
1982–1983	US (175)	Prospective control	100 (50)	45 (M), 44 (F)	Serositis	Neurological, alopecia, ↓ platelets	
1987–2012	US (multiethnic) (27)	Retrospective	1979 (7.9)	49.8 (M), 37.6 (F)	Hypertension, renal disease, Thrombotic episode, hypertension, disability, lymphopenia	Malar rash, RP, photosensitivity, oral ulcers, alopecia, arthralgia	Anti-Sm, DAT, LAC, anti-dsDNA, low C3 (M)
2002–2007	US (multiethnic) (195)	Case–control	265 (9)	NA	Proteinuria, lymphopenia, platelets count		6 antibodies assayed, *P* > 0.05

**Latin America**							

1997–2005	Latin America (176)	Inception	1213 (10.1)	27 (M), 29.2 (F)	Constitutional symptoms, hypertension, proteinuria, any renal, hemolytic anemia	Arthralgia, alopecia, RP, photosensitivity, any cutaneous	Low C3, IgG aCL (M)
1972–1993	Latin America (190)	Cross-sectional	1316 (8.1)	26 (M), 28 (F)	Renal disease	RP	dsDNA (M)
2000–2011	Colombia (multiethnic) (16)	Cross-sectional	160 (25)	32.0 (M), 30.5 (F)	Severe disease activity	Alopecia	anti-SSA/Ro (F)
2008–2012	Brazil (189)	Prospective	888 (8.1)	29.9 (M), 29.9 (F)	Malar rash, renal disease		Anti-dsDNA (M)

**Scandinavia, Europe, and North Africa**							

1980–1990	Spanish (186)	Prospective	261 (11.5)	34 (M), 31 (F)	Discoid lesion, subcutaneous lesion	Arthritis, malar rash	6 antibodies assayed, *P* > 0.05
1981–2000	Greek (184)	Retrospective	580 (14)	34.6 (M), 31.4 (F)	NA	Photosensitivity, RP, oral ulcers, anemia	NA
1982–2012	UK (multiethnic) (194)	Retrospective	484 (9.3)	30.9 (M), 29.1 (F)	NA	Oral ulcers	IgM aCL (F)
1987–2006	Spain (191)	Retrospective	150 (15.3)	54 (M) 43 (F)	Secondary Sjogren’s syndrome (over course of disease), thrombocytopenia	RP	Anti-SSA/Ro(F)
1989–2007	Greek (179)	Retrospective	743 (7.9)	34 (M), 31 (F)	Nephropathy, tendonitis, myositis	NA	NA
1990–1999	Tunisian (180)	Retrospective	295 (8.1)	NA	Vascular thrombosis	Alopecia	NA
1992–2006	Spain (182)	Retrospective	363 (13)	47.8 (M) 36.6 (F)	Serositis, renal disease, neurologic disorder	Leukopenia, alopecia	Anti-DNA (M)
2000–2008	Danish (199)	Retrospective	513 (11.5)	46.2 (M), 36.2 (F)	Serositis, nephropathy, hypertension	Photosensitivity	3 antibodies assayed, *P* > 0.05

**Middle East**							

1976–2011	Iran (188)	Retrospective	2355 (10.1)	25 (M), 24.5 (F)	Discoid rash, nephritis	Arthritis, leukopenia	
1996–2012	Turkey (185) (Mediterranean)	Retrospective	428 (6.8)	40.4 (M) 38.5 (F)	Renal disease, CNS	Dry eyes, Dry mouth, photosensitivity	

**Asia**							

1990–1993	Asian (192)	Retrospective	147 (41.5)	28.2 (M), NA (F)	NA	Arthritis, leukopenia	Anti-SSA/Ro (F)
1994–2010	Korea (196)	Retrospective	632 (9)	32.9 (M) 32.6 (F)	Renal disease	Discoid rash, alopecia, Leukopenia	Anti-SSA/Ro (F)
1999	HongKong (Asian) (32)	Retrospective control	252 (20.2)	31 (M), 31.9 (F)	NA	RP, alopecia	Anti-SSA/Ro (F)
2001	Malaysian (Asian) (193)	Prospective	134 (9.0)	30 (M), 26 (F)	Thrombosis	Arthritis	NA
2006–2010	Indian (Asian) (183)		250 (11.2)	22.3 (M), 28.3 (F)	Renal disease	Disease severity	Panel of 13 antibodies, *P* > 0.05
2008	Thai (Asian) (177)	Retrospective Case–control	111 (33.3)	34.6 (M), 34.4 (F)	↓ Platelets, ↑ Serum creatinine	Alopecia, arthralgia, RP, psychosis	7 antibodies assayed, *P* > 0.05
2010	Chinese (181)	Retrospective	1790 (9.8)	31.5 (M), 30.9 (F)	Serositis, pleuritis, and discoid rash	Malar rash, alopecia, oral ulcers, leukopenia positively correlates with age	Elevated ESR, antinuclear, anti-SSA/Ro and anti-SSB/La (F). Anti-SSB/La correlates with age (M)

*F, female; M, male; NA, not applicable; DAT, direct antiglobulin test; LAC, lupus anticoagulant; ESR, erythrocyte sedimentation rate; anti-dsDNA, anti-double stranded deoxyribonucleic acid; anti-Sm, anti-Smith; IgG Acl, anti-cardiolipin; anti-Sjögren’s syndrome-related antigen A (SSA/Ro), anti- Sjögren’s syndrome-related antigen B (SSB/La)*.

**Table 5:** Sex differences in ankylosing spondylitis clinical phenotypes.

Clinical phenotype	Studies showing phenotype is significantly higher in males	Studies showing phenotype is significantly higher in females	Studies showing statistically insignificant differences in males and females
**Baseline characteristics**			

Age at onset	(200)	(141, 201–204)	(19)^R(15)^, (205, 206)
Age at diagnosis		(141, 203, 204)	(19)^R(15)^, (202, 206)
Delay in diagnosis		(19)^R(15)^, (205)	(141, 203, 204, 206, 207)
Night pain		(206)	
Sleep disturbance		(206)	
Duration of morning stiffness		(207)	
Relevant family history		(200, 208, 209)	(202, 203)
HLA-B27–positive, %	(202–204, 209)		(19, 141, 200, 201, 205)

**Disease activity and functional index**			

ESR		(201, 206)	(141, 202, 203, 205, 207)
CRP	(203–205, 208)	(201)	(202, 206, 207)
Disease activity: BASDAI score		(201, 205–210)	(141, 202–204)
BAS-G		(208)	(203, 205)
Back pain	(201, 202, 209)	(203, 205)	
BASRI	(207, 209, 210)		
BASRI-spinal	(200, 201)		
BASRI-hip	(209)		
Physical function: BASFI score		(206, 208)	(141, 200, 201, 203–205, 207, 209, 210)
Spinal mobility: BASMI score	(203, 204, 207)	(202)	(205)
Occiput-to-wall distance	(202, 207, 209, 210)		
Chest expansion			(202, 203, 207, 209, 210)
Modified Schober’s test		(202, 209, 210)	(207)
Finger-to-floor	(203, 209, 210)		
Lumbar rotation		(203)	

**Clinical data**			

MASES		(204, 209)	
Enthesitis		(202–208, 210)	(141, 209)
Swollen joint score		(204, 208, 209)	(205, 210)
Tender joint score		(205, 207–209)	
Definite deterioration and radiographic progression—cervical spine		(208, 211)	(202)
Cervical pain			(203, 209)
Radiographic sacroiliitis, %	(208, 209)		
Dactylitis		(209)	(204, 210)
Root joint involvement (shoulder and hip)	(205)	(141)	(202, 203, 209)
Localization of clinical symptoms to buttock		(208)	
Peripheral arthritis		(204, 208)	(141)
Upper limb arthritis (%)		(209)	
Lower limb arthritis (%)			(209)
Knee involvement	(202)		(203)
Intensity of axial pain	(209)	(208)	
mSASSS	(205)		
Thoracic syndesmophyte	(202)	(203)	
Bamboo spine	(202)		
Definite deterioration and radiographic progression—lumbar spine	(211)		(202)
MRI-inflammatory lesions of the spine, %	(208)		(210)
Uveitis		(141, 202)	(203–205, 209)

**Measures of Quality of Life**			

SF-36 mental score	(205, 208)		
SF-36 physical score	(208)		(205)
ASQoL score		(209, 210)	(203, 205)
EuroQoL score			(205)
HAQ-AS		(208)	(200)

*The results depict studies where significant (*P* < 0.05) differences were detected; ^R(x)^ indicates meta-analysis of x number of published studies; ESR, erythrocyte sedimentation rate; CRP, C-reactive protein; BASDAI, Bath Ankylosing Spondylitis Disease Activity Index; BAS-G, Bath Ankylosing Spondylitis Patient Global disease activity score; BASRI, The Bath Ankylosing Spondylitis Radiology Index; BASFI, Bath Ankylosing Spondylitis Functional Index; BASMI, Bath Ankylosing Spondylitis Metrology Index; MASES, Maastricht Ankylosing Spondylitis Enthesitis Score; mSASSS, modified Stoke Ankylosing Spondylitis (AS) Spine Score; SF-36, Medical Outcomes Study 36-item Short Form; ASQoL, Ankylosing Spondylitis Quality of Life Questionnaire; EuroQoL, European Quality Of Life scale; HAQ-AS, Health Assessment Questionnaire for the Spondyloarthropathies*.

**Table 6:** Comparative studies of male and female ankylosing spondylitis: main clinical and demographic findings.

Year of Study	Country (ethnicity)	Study type	Size (% female)	Age at onset	Clinical data (*P* < 0.05)		Serology
						
					Higher in males	Higher in females	
**North America**							

2007	USA (White, African American, Asian/Pacific Islander, Native American, Hispanic, others) (200)	Prospective	402 (24.9)	23.6 (M), 21.5 (F)	BASRI, BASFI and HAQ-S (when adjusted for BASRI), thoracic, and lumbar spinal radiographic severity	AS family history, neck and peripheral joint pain^#^	NA

**Latin America**							

2006–2009	Brazil (209)	Prospective	1,505 (27.6)	NA	% of HLA-B27+ patients, axial inflammatory pain, lumbar pain, urethritis, occiput-to-wall and finger-to-floor distances, BASRI, BASRI-spine, BASRI-hip, grade 4 sacroiliitis	AS family history, upper limb arthritis, dactylitis and nail involvement, psoriasis, number of painful and swollen joints, MASES, BASDAI, ASQoL, Schober’s test	NA

2006	Argentina, Brazil, Costa Rica, Chile, Ecuador, Mexico, Peru, Uruguay, and Portugal (210)	Cross-sectional	1,072 (23.8)	NA	BASRI, occiput-to-wall and finger-to-floor distance	BASDAI, ASQoL, Enthesitis, Schober’s test	NA

**Europe**							

2004–2009	UK (206)	Prospective	516 (66.7)	NA		Night pain, sleep disturbance, BASDAI score, BASFI score	ESR (F)

2005–2016	Switzerland (204)	Prospective	440 (33.2)	25 (M), 27.3 (F)	% of HLA-B27+ patients, BASMI score	Diagnostic delay, peripheral arthritis, number of swollen joints, % enthesitis, MASES	CRP (M)

2004–2013	Spain (201)	Retrospective	1,514 (25.3)	26.7 (M), 28.2 (F)	Lumbalgia	AS family history	NA

2007–2010	France (208)	Prospective	475 (49.7)	NA	SF-36 mental and physical scores, % radiographic sacroiliitis, MRI-inflammatory lesion of sacroiliac joints and spine	Pain at cervical spine, buttock, axial, and peripheral joint pain intensity, tender joint and swollen joint scores, MASES, AS family history, BASDAI, BAS-G, BASFI scores, HAQ-AS, ASQoL	CRP (M)

1996–2008	Netherlands, Belgium, France (205)	Prospective	216 (62)	23.1 (M), 23.3 (F)	Hip involvement, SF-36 mental score, mSASSS	BASDAI, back pain, tender joint count, MASES	CRP (M)

**Middle East and North Africa**							

2010–2011	Iran (Fars, Turk, Kurd, Lor, and others) (203)	Prospective	320 (20.9)	22.2 (M), 24.3 (F)	% of HLA-B27+ patients, tragus-to-wall and finger-to-floor distances, BASMI, lateral lumbar flexion score	Enthesitis (thoracic, chest wall), elbow joint involvement, back pain, degree of lumbar rotation, lateral lumbar flexion distance, modified Schober’s test	CRP (M)

2009–2010	Morocco (207)	Prospective	130 (33.1)	27.9 (M), 28.8 (F)	Occiput-wall distance, BASMI, BASRI	Duration of morning stiffness, number of tender joints, BASDAI, Schober’s test, MEI	NA

**Asia**							

2009	India (141)	Prospective	70 (15.7)	22.3 (M), 30.0 (F)	NA	Uveitis, root joint involvement	NA

2006	Korea (202)	Cross-sectional	505 (14.1)	25.0 (M), 27.7 (F)	% of HLA-B27+ patients, joint pain, higher occiput-to-wall distance, thoracic syndesmophyte, bamboo spine	Uveitis, modified Schober’s test, knee joint involvement, plantar fasciitis	NA

*F, female; M, male; NA, not applicable; ESR, erythrocyte sedimentation rate; CRP, C-reactive protein; BASDAI, Bath Ankylosing Spondylitis Disease Activity Index; BAS-G, Bath Ankylosing Spondylitis Patient Global disease activity score; BASRI, The Bath Ankylosing Spondylitis Radiology Index; BASFI, Bath Ankylosing Spondylitis Functional Index; BASMI, Bath Ankylosing Spondylitis Metrology Index; MASES, Maastricht Ankylosing Spondylitis Enthesitis Score; mSASSS = modified Stoke Ankylosing Spondylitis (AS) Spine Score; SF-36 = Medical Outcomes Study 36-item Short Form; ASQoL = Ankylosing Spondylitis Quality of Life Questionnaire; HAQ-AS = Health Assessment Questionnaire for the Spondyloarthropathies; MEI = Mander enthesis index*.

## Results

### Males and Females Have Different Biological Landscapes

A report released by the Institute of Medicine in 2001, “Exploring the Biological Contributions to Human Health: Does Sex Matter?” describes in great detail the different factors that contribute to biological differences in males and females, and how these differences affect health and diseases in these two sexes (11). Beyond the overt differences in reproductive biology, males and females show differences in immune functions, brain organization, pain perception, gene dosing (for genes that escape X-chromosome inactivation) as well as metabolism, lifestyle, and physical performance, all of which may alter pharmacokinetic and pharmaco-dynamic variables in the two sexes (11). Males and females operate from different biological landscapes, be it in healthy, diseased, or recovering states.

To explain autoimmune sex dimorphism, it becomes necessary to first describe the cellular and hormonal interactions found in normal immune regulation and thereafter extrapolate these to autoimmune phenomena. In comparison to the innate immunity, the adaptive immune system is known to be significantly affected by sex. Adult females in general, show stronger immune responses than others, and these responses are partially modulated by sex hormones (3, 4, 6, 11). Other contributing parameters include genetic and environmental factors; we refer the readers to a string of excellent reviews for in-depth discussions of these factors (3–10). We summarize the sex dichotomy in normal immune system biology in Table 1 (3). All components of the innate immunity, such as the toll-like receptor pathway, antigen-presenting cells, dendritic cells, macrophages, granulocytes, and natural killer cells, show stronger activity in females. Despite the lower CD8+ T-cell count, the cytotoxicity of each of these cells is higher in females. As estrogen and progesterone levels wax and wane during the menstrual cycle, the balance between T_H_1 and T_H_2 system fluctuates. This balance and its interactions with other systems such as the T_H_17, T_reg_, and B-cells dictate the overall immune response. Disruptions to the equilibrium of these different systems lead to different AD pathologies and disease onsets in males and females (6).

### ADs Show Stronger Female Bias

**Figure 1:**
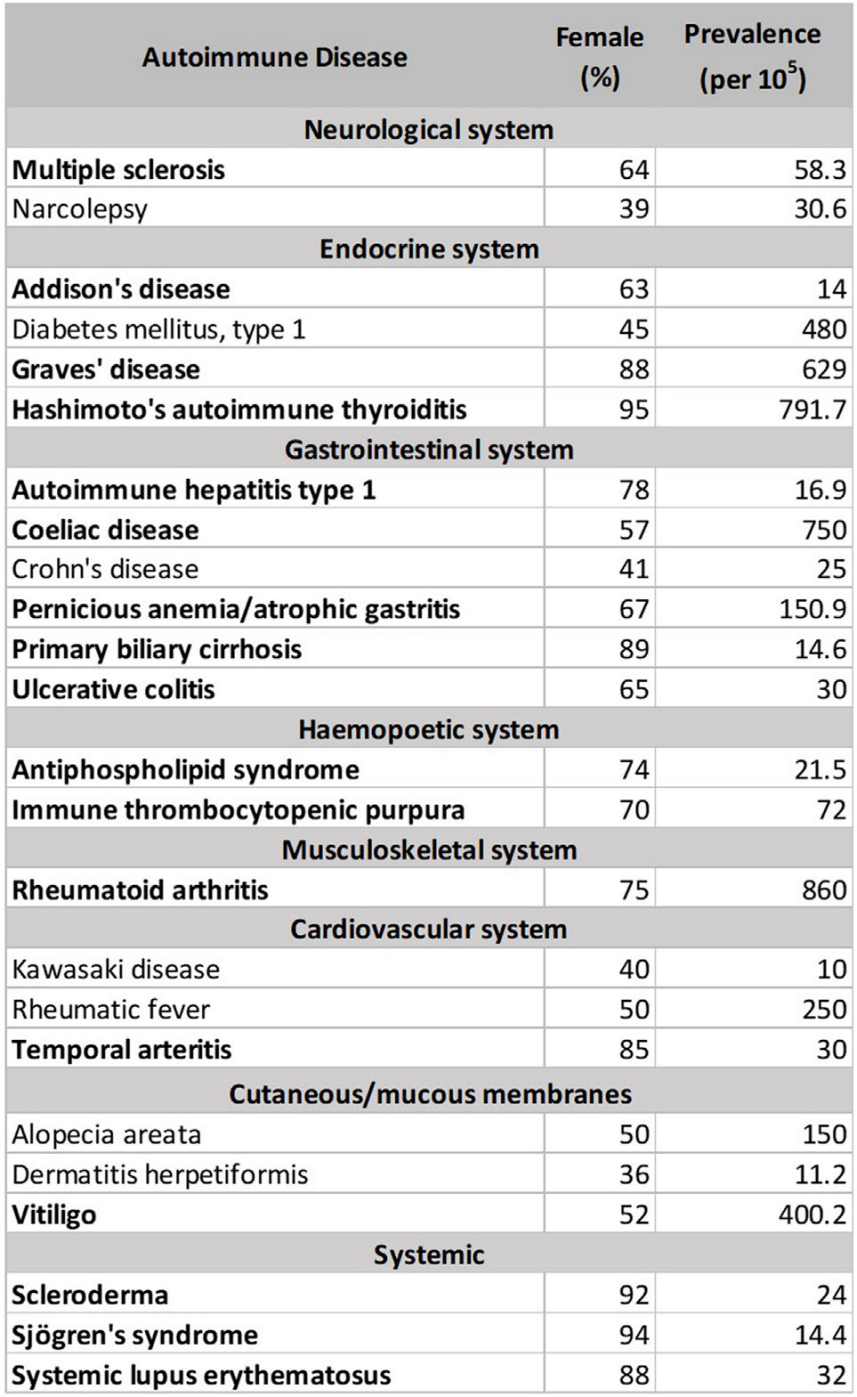
List of Autoimmune Diseases (ADs) with prevalence ≥ 1 per 10,000 people.

There are currently more than one hundred identified ADs, with 24 showing high prevalence (occurring in 1 per 10,000 people) (212). 71% of these common ADs (Figure 1 below, in bold) are more prevalent in females than males (>50% female prevalence), suggestive of a stronger female bias (213). However, these data were an aggregated one from “world,” and “USA” (213). In Table 2, we stratify some of these ADs by geographical location (as a proxy for ethnicity), in order to gain a better insight of each AD’s prevalence in different parts of the world. In addition, we include three ADs which have been reported to be more prevalent in males: idiopathic pulmonary fibrosis (IPF), ankylosing spondylitis (AS), and Guillain–Barré syndrome (GBS). Table 2 shows that female predominance prevails in all three systemic ADs. Regardless of the geography, females are more than thrice as likely as males to suffer from systemic ADs. For instance, Spanish women are 18.6 times more likely than men to suffer from Sjögren’s syndrome (SS). A similar trend is also observed with endocrine ADs: Grave’s disease and Hashimoto’s thyroiditis (HT). In India, the female to male ratio for HT is an astonishing 21.7 to 1. Another strong female bias (at least a 2:1 female to male ratio worldwide) is observed with some gastrointestinal and hepatic ADs (primary biliary cholangitis and autoimmune hepatitis), as well as a musculoskeletal AD (rheumatoid arthritis).

Although a strong female preponderance was observed with other ADs, these tend to vary with geographical locations. For example, antiphospholipid syndrome shows a female to male ratio of at least 3:1 worldwide, except in Spain and Italy, where it shows lower ratios (2.1:1 and 1.7:1, respectively). Immune thrombocytopenic purpura (ITP) was reported to have a 70% female prevalence in Denmark (213); however, the sex ratio is much lower in our dataset, ranging from 1.1:1 in the USA to 1.7:1 in France. Asian females seem to be more likely to get ITP than other ethnicities, with a female to male ratios ranging from 1.9:1 in India to 4.3:1 in Singapore. Other ADs reported include celiac disease (CoD) and ulcerative colitis (UC), which affected 57 and 65% of females, respectively (213). The female-to-male ratios for CoD and UC vary in our dataset, favoring males or females depending on geography. In Italy, UK, and India, males are more likely to suffer from CoD than females, whereas the reverse is true in Denmark and Sweden. In Asia, males are more likely to suffer from UC than in the Western world. AS and GBS show greater bias toward males, regardless of the geographical locations. In IPF, however, male predominance was observed all around the world except for the North Americas.

### ADs Have Different Trajectories in Different Sexes

Autoimmune diseases are quintessentially immune system disorders. As the biology of the immune system varies between the two sexes, one would expect ADs to have different disease trajectories in males and females. Here, we utilize a female-biased AD: systemic lupus erythematosus (SLE), as well as a male-biased AD: ankylosing spondylitis (AS) to exemplify sex-bias in disease progression, complications, and mortality. SLE and AS data are stratified by clinical data, geography, and period of study, since older studies may suffer from delayed diagnoses in male SLE and female AS patients (Tables 3–6).

In SLE, clinical phenotypes show sex-bias. Alopecia, photosensitivity, malar rash, Raynaud’s phenomenon, musculoskeletal complications, oral ulcers as well as psychiatric disorders are female-biased (Table 3). In addition, females are more likely to suffer from relapses and a concurrent diagnosis for SS. Male-specific clinical phenotypes include discoid lesions, serositis, renal disease, seizure, and peripheral neuropathy, as well as cardiovascular complications. Males also tend to display constitutional symptoms and higher score in Systemic Lupus Erythematosus Disease Activity Index, indicative of a higher disease activity. Indeed, higher mortality has been reported in male patients vs female patients. Serology in SLE also shows some sex-bias: females SLE patients more frequently present with higher erythrocyte sedimentation rate (ESR) and test positive for anti-SSA/Ro while the male SLE patients more frequently test positive for lupus anticoagulant, anti-Sm, anti-dsDNA, and hypocomplementemia.

In AS, clinical phenotypes also show some degree of sex-bias. Male AS patients tend to have disease onset at younger age and present with higher CRP, more back pain, knee involvement, higher scores for BASRI (including BASRI-spinal and BASRI-hip), radiographic sacroilitis, higher modified Stoke Ankylosing Spondylitis Spinal Score, lower functional indices (occiput-to-wall and finger-to-floor distances), but higher SF-36 mental and physical scores. Female AS patients, on the other hand, present more with AS family history, higher Bath Ankylosing Spondylitis Disease Activity Index (BASDAI) score, enthesitis, more numbers of swollen, tender joints and peripheral arthritis but higher ASQoL score. Notably, in a multivariate model, Lee et al. found that for a given level of radiographic damage, female AS patients have more functional limitations than their male counterparts (200).

## Discussion

In this systematic review, we summarize sex differences in immune system biology, AD prevalence, as well as clinical phenotypes of SLE and AS. Data accrued highlight female predominance in common ADs, although there exist geographical differences in some cases. These observed geographical differences are suggestive of potential contributions of genetics and environmental factors toward AD pathology.

In SLE, disease complications and serology seem to show sex-bias. Alopecia, for example, is exclusively observed in females just as serositis is exclusively seen in males. It is interesting how the serology in these males and females is reflective of sex-bias in clinical phenotypes. In The Genetic Profile Predicting the Phenotype (PROFILE) multiethnic cohort of 2,322 SLE patients, anti-Sm were significantly associated with antinuclear antibody, anti-double-stranded DNA (dsDNA), and clinical phenotypes, such as serositis, renal involvement, psychosis, vasculitis, Raynaud’s phenomenon, hemolytic anemia, leukopenia, lymphopenia, and arterial hypertension (214). Furthermore, double positive serology for anti-Sm and anti-dsDNA has been strongly associated with renal involvement (215–218) and higher disease activity (219). Most of these clinical phenotypes are male-specific SLE complications (Tables 3 and 4). Similarly, anti-SSA/Ro antibodies have been reported to be strongly associated with low C3 (hypocomplementemia) and clinical phenotypes such as photosensitivity, subacute cutaneous lupus erythematosus, cutaneous vasculitis (palpable purpura), hematological disorder (anemia, leukopenia, and thrombocytopenia) (220–227), as well as Jaccoud’s arthropathy (a type of arthritis) (228, 229). These phenotypes show female bias in our dataset (Tables 3 and 4). There are some clinical phenotypes such as mucocutaneous and hematological involvements, vasculitis, and association of anti-SSA/Ro with low C3 that differ between these correlation studies and our dataset. This may arise either from ethnic or age differences in the different study groups, or the size of the study groups. The trend for renal involvement persists in all of the studies we have analyzed; however, this clinical phenotype may or may not show a statistical difference for sex-bias owing to the low number of male SLE patients in some studies.

We also observed sex-bias in our dataset for AS clinical phenotypes: female AS patients present with enthesitis and higher BASDAI scores, while male patients present with higher BASRI scores. AS is clinically tested with HLA-B27, ESR, and CRP. While some studies suggested that high CRP is more significantly seen in male patients and high ESR with female patients, many other studies have not come to similar conclusions.

The findings from SLE and AS suggest that disease phenotypes differ between males and females. In some cases, these diseases arguably have higher activity in the sex having lower prevalence. Awareness of sex-bias in disease presentation is crucial for early diagnosis, as well as treatment strategies for ADs in different sexes. More importantly, such awareness may guide the development of improved study design strategies for biomarker discovery.

## Future Direction and Conclusion

Timely diagnosis and treatment can be very effective for AD patients (230, 231) and biomarkers have great potential to enable it. Although AD biomarkers discovery is thriving, the same cannot be said of their clinical translation. Many biomarker projects fail at validation/replication stage (13) due to suboptimal sensitivity and specificity, as well as reproducibility in different studies (12). A few potential contributing factors to this observed failure include suboptimal infrastructure, study design, and execution in discovery-phase (12). Suboptimal study design includes small sample numbers, lack of patient history and subject matching (in terms of age, race, and sex) (12). Here we highlight the importance of sex stratification in biomarker discovery studies to promote reproducibility in replication/validation stage. Drawing example from SLE and AS, we note that differential clinical phenotypes exist in male and female patients. Different sexes may require different biomarkers for proper diagnosis of the same disease. From SLE serology we learn that some biomarkers are more frequently detected in one specific sex, and they show strong associations with sex-biased clinical phenotypes. Such specific associations may be missed when data from both sexes are aggregated.

In addition to enhancing sex-specific biomarker discovery and promoting reproducibility, a thorough understanding of sex differences in autoimmune milieu may guide disease prevention, diagnosis, and management. Our findings in Table 2 clearly demonstrate a higher prevalence ADs among females. Breast cancer screening mammography among women at average risk aged 50–74 has been shown to reduce breast cancer mortality by 30–40% (232). These findings suggest potential benefits of AD screenings specifically for women, for early AD detection and reduction of mortality rates through early intervention. Another plausible area of further study is to sex-stratify serological benchmarks for males and females, in light of varying cytokine levels and activity in different sexes as observed in Table 1. We have limited our scope in this review to SLE, AS and sex stratification. Further stratifications for improved patient segmentation and more specific biomarker discovery may include stratifications by age, ethnicities and disease stages.

## Author Contributions

KP conceptualized, gathered literature for all other autoimmune diseases, consolidated literature review from others, and wrote the manuscript. JO gathered literature for IPF, SLE, gastrointestinal and liver autoimmune diseases and proofread the manuscript. SD gathered literature for autoimmune hepatitis. WT gathered literature for rheumatoid arthritis. NM gathered literature for ankylosing spondylitis and proofread the manuscript. JL gathered literature for SLE and antiphospholipid syndrome. CLD contributed to study design and provided clinical insights which enhanced manuscript quality.

## Conflict of Interest Statement

The authors declare that the research was conducted in the absence of any commercial or financial relationships that could be construed as a potential conflict of interest. The reviewer YL and handling Editor declared their shared affiliation.
