# Supplementary material for: The Importance of Sex Stratification in Autoimmune Disease Biomarker Research: A Systematic Review
Source: Front Immunol. 2018 Jun 4;9:1208. doi: 10.3389/fimmu.2018.01208 (PMC5994590; doi:10.3389/fimmu.2018.01208)
Supplement: Supplementary file 1 [file data_sheet_1.DOCX]

**Supplementary Table 1. Biomarkers for autoimmune diseases (ADs)**

| **Disease name** | **Diagnosis method** | **Biomarkers** |
| --- | --- | --- |
| **Systemic and connective tissue** | | |
| Systemic Lupus Erythematosus (SLE) | - SELENA-SLEDAI: Safety of Estrogens in Lupus Erythematosus National Assessment (SELENA)-Systemic Lupus Erythematosus Disease Activity Index (SLEDAI) - British Isles Lupus Activity Group (BILAG) - Systemic Lupus International Collaborating Clinics (SLICC) criteria[^1^](#_ENREF_1) | Antibodies:   - Anti-Ribonuclease H2 (RNase H2)[^2^](#_ENREF_2) - Anti-proliferating cell nuclear antigen protein (PCNA) - Anti-double stranded DNA (dsDNA) - Anti-nuclear antibody (ANA) - Anti-U1A, anti-U2B - Anti-aldolase A[^3^](#_ENREF_3): SLE associated with nephritis - Anti-ezrin[^4^](#_ENREF_4) and anti-annexin A2[^4^](#_ENREF_4)^,^ [^5^](#_ENREF_5): SLE with proliferative lupus nephritis complication - Anti-Smith |
|  |  | Cytokines:   - Type I interferon (IFN) signature - Interleukin 12, 17, 18[^6^](#_ENREF_6)^,^ [^7^](#_ENREF_7) - IL-18/IL-4 ratio[^7^](#_ENREF_7) |
|  |  | Other candidate biomarkers[^8^](#_ENREF_8):   - Apolipoprotein CIII[^9^](#_ENREF_9): SLE with lupus nephritis complication - Serotransferrin[^10^](#_ENREF_10): correlates with SLE disease activity - sTNF-R2 |
| Sjögren’s syndrome (SS) | - European League Against Rheumatism (EULAR) SS disease activity index (ESSDAI) scores - EULAR Sjögren’s syndrome patient reported index (ESSPRI) - SS disease damage index (SSDDI) - Schirmer test - Salivary gland scintigraphy | Antibodies:   - Anti-nuclear antibody (ANA) as validation instead of screening - Anti-Sjögren's syndrome-related antigen A [SS-A (Ro)] - Anti- Sjögren's syndrome-related antigen B [SS-B (La)] - Anti-salivary gland protein 1 (SP-1), anti-carbonic anhydrase 6 (CA6) and anti-parotid secretory protein (PSP[^11^](#_ENREF_11)): for early identification of primary SS - IgM rheumatic factor (anti-IgG Fc) |
|  |  | Cytokines:   - Type I IFN signature, e.g. MxA^[12](#_ENREF_12)^ at cutoff 100 μg/L - CXCL13, TNF-R2, and CD48[^13^](#_ENREF_13) |

| **Disease name** | **Diagnosis method** | **Biomarkers** |
| --- | --- | --- |
| **Skin** | | |
| Scleroderma (systemic sclerosis, SSc) | - Semi-quantitative assessment of skin thickness (modified Rodnan skin score [mRSS]) | Antibodies:   - Anticentromere (ACA)[^14^](#_ENREF_14): related to limited SSc (CREST syndrome), observed more in older female caucasians^[15](#_ENREF_15)^ - Anti-Scl-70 or anti-topoisomerase (TOPO)[^16^](#_ENREF_16): related to diffuse SSc and pulmonary fibrosis, observed more in African-American patients[^15^](#_ENREF_15) - Anti-RNA Polymerase III (Pol 3)[^16^](#_ENREF_16): associated with diffuse SSc and renal crisis[^15^](#_ENREF_15) - Anti-Th/To (Th/To)[^16^](#_ENREF_16): associated with limited SSc - Anti-fibrillarin or anti-U3-RNP (U3- RNP), anti-U1-RNP (U1-RNP): associated with diffuse SSc, observed more in African-American patients[^15^](#_ENREF_15) - Anti-PM/Scl (PM/Scl): associated with polymyositis and SSc^[14](#_ENREF_14)^   Cytokines:   - Soluble IL2 receptor (srIL-2)[^17^](#_ENREF_17)^,^ [^18^](#_ENREF_18) - CXCL4: correlated with skin, lung fibrosis and pulmonary arterial hypertension; predicts SSc risk and progression[^19^](#_ENREF_19)   Organ specific:   - Vascular, fibrosis, pulmonary hypertension and pulmonary fibrosis: Please refer to [^20^](#_ENREF_20)^,^ [^21^](#_ENREF_21) |
| Psoriasis | - Psoriasis Area Severity Index (PASI) score | Cytokines:   - IL17A and IL17F[^22^](#_ENREF_22) - TNF- α, IFN-γ, IL-6, IL-8, IL-12 and IL-18[^23^](#_ENREF_23) - IL22[^24^](#_ENREF_24)   Other candidate biomarkers:   - Desmoplakin, complement C3, polymeric immunoglobulin receptor, and cytokeratin 17: correlated with PASI[^25^](#_ENREF_25) - Galectin 3 binding protein (G3BP)[^26^](#_ENREF_26) - Cytoskeletal, calcium-binding proteins and their peptides: thymosin β4, talin 1, actin γ, filamin, prolifin, cytoskeletal, calgranulins A and B[^27^](#_ENREF_27) - C-reactive protein (CRP), platelet P-selectin[^28^](#_ENREF_28), haptoglobin, complement component 3 (C3), and C4[^29^](#_ENREF_29) - Serum leptin, resistin and lipocalin[^30^](#_ENREF_30) |

| **Disease name** | **Diagnosis method** | **Biomarkers** |
| --- | --- | --- |
| **Pulmonary** | | |
| Idiopathic pulmonary fibrosis (IPF) | - Official ATS/ERS/JRS/ALAT Statement: Idiopathic Pulmonary Fibrosis guidelines[^31^](#_ENREF_31) | Cytokine:   - IL-8[^32^](#_ENREF_32)^,^ [^33^](#_ENREF_33): worse prognosis (>7.2 pg/ml) - CXCL13   Other candidate biomarkers:   - Krebs von den Lungen-6 Antigen (KL-6)[^34^](#_ENREF_34):   >1000 U/ml for worse prognosis; >1300 U/ml increased risk of acute exacerbation   - Surfactant proteins A (SP-A) and D (SP-D)[^35-37^](#_ENREF_35) - Matrix Metalloproteinase-1 (MMP1) and MMP7[^38-41^](#_ENREF_38) |
| **Hematopoetic and vascular** | | |
| Antiphospholipid syndrome | - One clinical criteria (Vacular thrombosis or Pregnancy morbidity) and - One laboratory criteria (LA, aCL and/or anti-β2GPI)[^42^](#_ENREF_42) | Antibodies:   - Lupus anticoagulant (LAC)[^43^](#_ENREF_43) - Anticardiolipin (aCL)[^44^](#_ENREF_44) - Anti-β2 glycoprotein-I (anti-β2GPI )[^45^](#_ENREF_45) - Anti-phosphatidylserine/prothrombin (aPS/PT)[^46^](#_ENREF_46) |
| Immune thrombocytopenic purpura (ITP) | - Review of peripheral smear and evaluation of history and examination of the patient[^47^](#_ENREF_47) | Antibodies:   - Glycoprotein (GP) specific autoantibodies (e.g., GPVI, GPIb/IX, GPIIb/IIIa autoantibodies)[^48^](#_ENREF_48)^,^ [^49^](#_ENREF_49) - Anti-ADAMTS13   Ctyokines:   - B-lymphocyte activating factor (BAFF)[^50^](#_ENREF_50) - A proliferation-inducing ligand (APRIL)[^51^](#_ENREF_51) - CXCL10[^52^](#_ENREF_52) - CX3CL1 and IL-22: in pediatric ITP[^53^](#_ENREF_53) |

| **Disease name** | **Diagnosis method** | **Biomarkers** |
| --- | --- | --- |
| **Endocrine** | | |
| Grave’s disease | - Combination of eye signs, goiter, and any of the characteristic symptoms and signs of hyperthyroidism[^54^](#_ENREF_54) | Antibodies:   - Anti-thyroid antigens: thyroglobulin, thyroid peroxidase, sodium-iodide symporter and the thyrotropin receptor, thyroid-stimulating hormone receptor, thyroid-Stimulating Immunoglobulin (TSIg), thyrotropin Binding Inhibitory Immunoglobulins (TBII)[^55^](#_ENREF_55)^,^ [^56^](#_ENREF_56) - Anti-immunoglobulin G4[^57^](#_ENREF_57)   Cytokines:   - B-lymphocyte activating factor (BAFF)[^58^](#_ENREF_58) - CCL20[^59^](#_ENREF_59) - IL4, IL6, IL10[^60^](#_ENREF_60) - IP-10 (CXCL10/interferon-γ-inducible protein 10)[^61^](#_ENREF_61) - Soluble CD28[^62^](#_ENREF_62) - Interleukin 33 (IL33)[^63^](#_ENREF_63)   Other candidate biomarkers:   - Endothelin-1[^64^](#_ENREF_64) |
| Hashimoto’s thyroiditis | - Demonstration of circulating antibodies to thyroid antigens (mainly thyroperoxidase and thyroglobulin) and reduced echogenicity on thyroid sonogram in a patient with proper clinical features[^65^](#_ENREF_65)^,^ [^66^](#_ENREF_66) | Antibodies:   - Anti-thyroid antigens: thyroglobulin, thyroid peroxidase[^56^](#_ENREF_56) - Anti-immunoglobulin G4[^67^](#_ENREF_67)   Cytokines:   - B-lymphocyte activating factor (BAFF) - IP-10 (CXCL10/interferon-γ-inducible protein 10)[^61^](#_ENREF_61) - Reduced IL35[^68^](#_ENREF_68)   Other candidate biomarkers:   - Endothelin-1[^64^](#_ENREF_64) - Parathyroid hormone[^69^](#_ENREF_69) |
| Type 1 diabetes (adult*) | - Diagnosis and Classification of Diabetes Mellitus Report criteria[^70^](#_ENREF_70)^,^ [^71^](#_ENREF_71) | Antibodies:   - Anti-islet cells (ICA): islet cell antibody 512 (ICA512) - Anti-tyrosine phosphatases IA-2 and IA-2β[^72^](#_ENREF_72)^,^ [^73^](#_ENREF_73) - Anti-insulin (IAA) - Anti-glutamic acid decarboxylase (GAD65)[^72^](#_ENREF_72) - Anti-Zinc transporter 8 (ZnT8A)[^74^](#_ENREF_74): used in combination with anti-GAD, anti-IA2 and IAA to detect 98% of T1D autoimmune reactive indivicuals^[75](#_ENREF_75)^ - Antiaminoacyl-tRNA synthetase (aaRS)[^76^](#_ENREF_76)   Cytokines:   - Chemokine ligand 1 (CXCL1)[^77^](#_ENREF_77)   Other candidate biomarkers:   - Serum amyloid protein A (SAA) and C-reactive protein (CRP), as well as adiponectin and insulin-like growth factor binding protein 2[^78^](#_ENREF_78) |

| **Disease name** | **Diagnosis method** | **Biomarkers** |
| --- | --- | --- |
| **Gastro-intestinal and liver** | | |
| Primary biliary cholangitis | - Two of the following three criteria are satisfied[^79^](#_ENREF_79)^,^ [^80^](#_ENREF_80):  1. Anti-mitochondrial antibodies (AMA) titer > 1:40; 2. Alkaline phosphatase (AP) > 1.5 the normal upper limit for > 24wks 3. Liver histology showing nonsuppurative destructive cholangitis and interlobular bile duct destruction | Antibodies:   - Anti-mitochondrial antibodies (AMA) targeting the 2-oxo-acid dehydrogenase complexes[^81^](#_ENREF_81) - Antinuclear antibodies (ANA) targeting dsDNA, Sm, chromatin, ribosomal-P, RNP, SmRNP, SSA, SSB, and centromere)[^82^](#_ENREF_82)   - Anti-promyelocytic leukemia protein (PML) nuclear body (NB): anti-Sp140, anti-Sp100 and anti-PML antibodies[^83^](#_ENREF_83) - Thrombophilia-associated autoantibodies (i.e. anti-beta2GPI, phosphatydilserine, prothrombin)[^82^](#_ENREF_82)   Cytokines:   - IL18[^84^](#_ENREF_84) - TNF-α[^85^](#_ENREF_85) - IFN-γ and IL-5[^86^](#_ENREF_86) |
| Autoimmune hepatitis type I | - Scoring system based on a combination of clinical, serological, and histological criteria[^87^](#_ENREF_87) | Antibodies[^88^](#_ENREF_88):   - Anti-smooth muscle antibodies (SMA):[^89-91^](#_ENREF_89)   - Anti-filamentous actin antibodies (A-FAA)[^90^](#_ENREF_90)   - Anti-intermediate filaments (vimentim, desmin, Skeltin) - Anti-nuclear antibodies (ANA): e.g. antibodies to histones, double stranded RNA, chromatin, ribonucleoproteins, ribonucleoprotein complexes[^89^](#_ENREF_89)^,^ [^91^](#_ENREF_91)^,^ [^92^](#_ENREF_92) - Atypical perinuclear anti-neutrophil cytoplasmic antibodies (pANCA)[^93^](#_ENREF_93) - Autoantibodies to asialoglycoprotein receptor[^89^](#_ENREF_89): for seronegative ANA/SMA - Anti-soluble liver antigen (SLA), anti-soluble liver pancreas (SLP)[^92^](#_ENREF_92): for seronegative ANA/SMA   Other candidate biomarkers:   - Complement (C3) and alpha-2-macroglobulin (A2M)[^94^](#_ENREF_94) - Adiponectin[^95^](#_ENREF_95) - Gamma globulin[^87^](#_ENREF_87) |
| Ulcerative colitis | - Endoscopy, followed by confirmatory biopsy specimens[^96^](#_ENREF_96) - The Modified Mayo Endoscopic Score (MMES)[^97^](#_ENREF_97) - Ulcerative Colitis Endoscopic Index of Severity (UCEIS)[^98^](#_ENREF_98) | Antibodies:   - Antineutrophil cytoplasmic antibodies (ANCAs)[^99^](#_ENREF_99)^,^ [^100^](#_ENREF_100) - Serum anti-p53 antibodies (p53Abs)[^101^](#_ENREF_101)   Cytokines:   - IL-23[^102^](#_ENREF_102), IL-8[^103^](#_ENREF_103) - Soluble ST2 (sST2)[^104^](#_ENREF_104)   Other candidate biomarkers:   - Leucine-rich alpha-2 glycoprotein (LRG)[^105^](#_ENREF_105) - Alpha -1 antitrypsin (AAT) and granulocyte colony-stimulating factor (G-CSF)[^106^](#_ENREF_106) - Serum C-reactive protein (CRP)[^107^](#_ENREF_107)^,^ [^108^](#_ENREF_108) - Serum Human Trefoil Factor 3[^109^](#_ENREF_109) |
| Crohn’s disease | - Crohn’s Disease Activity Index (CDAI)[^110^](#_ENREF_110)^,^ [^111^](#_ENREF_111) - Combination of endoscopic, histological, radiological, and/or biochemical investigations[^112^](#_ENREF_112) | Antibodies:   - Anti-Saccharomyces cerevisiae antibody (ASCA IgA)[^113^](#_ENREF_113) - Anti-laminaribioside (ALCA), anti-mannobioside (AMCA) and anti-chitobioside (ACCA) antibodies [^114^](#_ENREF_114) - Anti-synthetic mannoside antibodies (AΣMA)[^115^](#_ENREF_115) - anti-OmpC, or anti-I2[^116^](#_ENREF_116)   Cytokines:   - **TNF-α , IL-12P40* ,** EGF, FGF2, eotaxin-1, IFN-α2, MDC (CCL22), IL-13, IL-5, IL-1α, MCP-1, MIP-1α, and VEGF[^117^](#_ENREF_117)   ** Markers used as therapeutic agents in clinics*  Other candidate biomarkers:   - Serum calprotectin[^118^](#_ENREF_118) - YKL-40, also known as Chitinase-3-like protein 1 (CHI3L1)[^119^](#_ENREF_119) |
| Coeliac disease (adult*) | - Serologic testing of celiac-specific antibodies and histopathologic examination of duodenal mucosal biopsy on a gluten-containing diet[^120^](#_ENREF_120)^,^ [^121^](#_ENREF_121) [^122^](#_ENREF_122) | Antibodies:   - Antihuman tissue transglutaminase (anti-tTG)[^123^](#_ENREF_123) - Endomysium antibody (EMA)[^124-126^](#_ENREF_124) - Deamidated gliadin peptides (DGP) antibodies[^127^](#_ENREF_127) - Anti-gliadin[^55^](#_ENREF_55)   Cytokines:   - Th-1, Th-2 and primarily APC derived cytokines[^128^](#_ENREF_128)   Other candidate biomarkers:   - Intestinal-fatty acid binding protein (I-FABP)[^129^](#_ENREF_129) |

| **Disease name** | **Diagnosis method** | **Biomarkers** |
| --- | --- | --- |
| **Musculoskeletal** | | |
| Ankylosing spondylitis | - Test for HLA-B27 - Inflammation imaging by MRI - Erythrocyte sedimentation rate (ESR) - X-ray - Bath Ankylosing Spondylitis Disease Activity Index (BASDAI)[^130^](#_ENREF_130) - Bath ankylosing spondylitis functional index (BASFI)[^131^](#_ENREF_131) - AS Disease Activity Score (ASDAS)[^132^](#_ENREF_132) - ASsessment in Ankylosing Spondylitis International Working Group criteria [ASAS20] | Antibodies:   - Anti-CD74[^133^](#_ENREF_133)^,^ [^134^](#_ENREF_134) - Anti-cxtracellular matrix proteins (connective tissue growth factor, glypican 3 and 4, matrix Gla protein, secreted modular calcium-binding protein 1 or SMOC1)[^135^](#_ENREF_135) - Anti- Bone remodeling factors (chondromodulin, purinergic receptor P2RX7, melanocortin 4 receptor, osteoglycin, osteonectin)[^135^](#_ENREF_135) - Anti- Protein phosphatase magnesium-dependent 1A (PPM1A)[^136^](#_ENREF_136)   Cytokines:   - IL23 - IL1[^137^](#_ENREF_137) - IL6[^138^](#_ENREF_138)^,^ [^139^](#_ENREF_139) - IL17[^140^](#_ENREF_140) - TNF alpha[^141-145^](#_ENREF_141)   Other candidate biomarkers:   - and ERAP1 - Vascular endothelial growth factors (VEGF)[^137^](#_ENREF_137)^,^ [^138^](#_ENREF_138) - C-reactive protein (CRP)[^137^](#_ENREF_137)^,^ [^146^](#_ENREF_146) - Matrix metallo protein (MMP-3)[^139^](#_ENREF_139) - Phosphodiesterase 4[^147^](#_ENREF_147) - Intracellular adhesion molecule (ICAM-1), Vascular cell adhesion molecule (VCAM-1)[^137^](#_ENREF_137) |
| Rheumatoid arthritis | - Disease Activity Score (DAS) 28 and erythrocyte sedimentation rate (ESR)[^148^](#_ENREF_148) - 2010 rheumatoid arthritis classification criteria[^149^](#_ENREF_149) - Patient Activity Scale (PAS) or PASII[^150^](#_ENREF_150) - Routine Assessment of Patient Index Data (RAPID3)[^151^](#_ENREF_151) - Clinical Disease Activity Index (CDAI) - Simplified Disease Activity Index (SDAI)[^152^](#_ENREF_152) and - Others[^153^](#_ENREF_153) | Antibodies:   - Anti–cyclic citrullinated peptide antibodies IgG isotype (IgG anti-CCP)[^154^](#_ENREF_154) - Antinuclear antibody (ANA)[^154^](#_ENREF_154) - Anti-CD26 isotypes[^155^](#_ENREF_155) - Immunoglobulin M rheumatoid factor (IgM-RF)[^156^](#_ENREF_156) - Anti-collagen II[^55^](#_ENREF_55)   Cytokines:   - IL-6[^154^](#_ENREF_154)^,^ [^157^](#_ENREF_157) - TNF-alpha[^154^](#_ENREF_154) - Serum 14-3-3η[^158^](#_ENREF_158)   Other candidate biomarkers:   - Calcium binding proteins (Calgranulin A, B and C)[^159^](#_ENREF_159) - C-reactive protein[^154^](#_ENREF_154)^,^ [^159^](#_ENREF_159) - Thymosin beta 4[^160^](#_ENREF_160), actin, tubulin, vimentin[^159^](#_ENREF_159) - Survivin^[157](#_ENREF_157)^ - Calprotectin[^161^](#_ENREF_161) |

| Psoriatic arthritis | - Bath Ankylosing Spondylitis Disease Activity Score (BASDAI)[^130^](#_ENREF_130) - Modified American College of Rheumatology (ACR20) response criteria - Psoriasis Area and Severity Index (PASI) | Antibodies:   - Anti-agalactosyl IgG[^162^](#_ENREF_162) - Antibodies against citrullinated proteins/peptides (ACPA) especially anti-mutated citrullinated vimentin (anti-MCVs)[^163^](#_ENREF_163) - Anti-cyclic citrullinated peptide antibodies (anti-CCP)[^164^](#_ENREF_164)   Cytokines:   - IL2, IL10 24, 25 - IL6[^165^](#_ENREF_165) - C-reactive protein[^165^](#_ENREF_165)^,^ [^166^](#_ENREF_166) - Soluble interleukin-2 receptor alpha (IL-2Rα 42)[^165^](#_ENREF_165) - CD5-like protein (CD5L)[^167^](#_ENREF_167) - Plasma YKL-40[^168^](#_ENREF_168)   Other candidate biomarkers:   - Osteoprotegerin^[166](#_ENREF_166)^ - Matrix metalloproteinase 3 (MMP-3)[^166^](#_ENREF_166) - C-propeptide of Type II collagen (CPII) to collagen fragment neoepitopes Col2-3/4(long mono)(C2C) ratio [CPII:C2C ratio][^166^](#_ENREF_166) - Dkk-1 and M-CSF[^169^](#_ENREF_169) - Integrin-beta 5 (ITGB5)[^167^](#_ENREF_167) - Mac-2-binding protein (M2BP)[^167^](#_ENREF_167) - Myeloperoxidase (MPO)[^167^](#_ENREF_167) |
| --- | --- | --- |

| **Disease name** | **Diagnosis method** | **Biomarkers** |
| --- | --- | --- |
| **Neurological** | | |
| Multiple sclerosis | - The 2010 revisions to the McDonald Criteria[^170^](#_ENREF_170) - Kurtzke Expanded Disability Status Scale (EDSS)[^171^](#_ENREF_171) - MS Severity Scale (MSSS) - MS Functional Composite (MSFC) | Antibodies[^172^](#_ENREF_172):   - IgM antibodies against Glc(alpha1,4)Glc(alpha) (GAGA4): in relapsing remitting multiple sclerosis (RRMS)[^173^](#_ENREF_173) - Autoantibodies against paranodal and axonal proteins (e.g. anti-Neurofascin155 and anti-contactin2)[^174^](#_ENREF_174) - Autoantibodies against oligodendrocytic and astrocytic proteins (e.g anti- inward-rectifying glial potassium channel KIR4.1)[^175^](#_ENREF_175) - Autoantibodies against myelin proteins and heat shock proteins (HSPs) (e.g. Proteolipid protein – PLP, and HSP70)[^176^](#_ENREF_176) - Anti- myelin oligodendrocyte glycoprotein (anti-MOG) - Anti-proteolipid protein   Cytokines[^177^](#_ENREF_177):   - IL23[^178^](#_ENREF_178) - CXCR3 - IFN‑γ and IL6, IL12 and IL4 levels: higher in RRMS patients[^179^](#_ENREF_179) - TNFα, IL10 and IL17: lower in active RRMS vs inactive RRMS - IL2, IL13[^180^](#_ENREF_180)   Other candidate biomarkers:   - Combination of HGF, Eotaxin/CCL11, EGF and MIP-1β/CCL4: for discriminating a dichotomous RR-MS versus progressive form (SPMS – secondary progressive MS and PPMS – primary progressive MS)[^181^](#_ENREF_181) - Pentosidine: advanced glycation endproducts (AGEs) as inflammatory biomarker in MS[^182^](#_ENREF_182) - Pentraxin3 (PTX3)[^183^](#_ENREF_183) |
| Myasthenia gravis | - Myasthenia gravis: Association of British Neurologists’ management guidelines[^184^](#_ENREF_184) | Antibodies[^185^](#_ENREF_185):   - Anti–acetylcholine receptor (anti-AChR)[^186^](#_ENREF_186) - Anti-muscle specific kinase (anti-MuSK)[^186^](#_ENREF_186) - Anti-low-density lipoprotein receptor-related protein 4 (anti-LRP4)[^185^](#_ENREF_185) - Anti-agrin[^187^](#_ENREF_187)^,^ [^188^](#_ENREF_188), anti-titin[^189^](#_ENREF_189) - Anti-KV1.4[^190^](#_ENREF_190) - Anti-ryanodine receptors[^191^](#_ENREF_191) - Anti-collagen Q[^192^](#_ENREF_192), Anti-cortactin[^193^](#_ENREF_193)   Cytokines:   - Resistin[^194^](#_ENREF_194) - CXC chemokine ligand 13 (CXCL13): associated with thymic lymphoid hyperplasia[^195^](#_ENREF_195)   Other candidate biomarkers:   - Matrix metalloproteinase 10 (MMP-10)[^196^](#_ENREF_196) - C-X-C motif ligand 1 (CXCL1)[^196^](#_ENREF_196) - Brain derived neurotrophic factor (BDNF)[^196^](#_ENREF_196) - Transforming growth factor alpha (TGF-α)[^196^](#_ENREF_196) |
| Guillain Barré syndrome | - Diagnostic criteria for GBS[^197^](#_ENREF_197)^,^ [^198^](#_ENREF_198) - Brighton Collaboration new case definitions for GBS[^199^](#_ENREF_199) - Lumbar puncture and nerve conduction studies[^198^](#_ENREF_198) - GBS Disability Scale[^200^](#_ENREF_200) - The Rasch-built Overall Disability Scale[^201^](#_ENREF_201) - Medical Research Council (MRC) sum score - The new Rasch-built MRC score to measure muscle[^202^](#_ENREF_202) strength[^203^](#_ENREF_203) | Antibodies[^198^](#_ENREF_198):   - Anti-peripheral myelin proteins (PMP22[^204^](#_ENREF_204), P2_14–25_,[^205^](#_ENREF_205)) - Anti-GA1, anti-ganglioside GM1a, GM1b, GD1a, GalNAc-GD1a: to detect acute motor axonal neuropathy (AMAN) - Anti-GM1, anti-GD1a: to detect acute motor sensory axonal neuropathy (AMSAN) - Anti-GT1a: to detect pharyngeal–cervical brachial variant - Anti-GQ1b (to detect Miller Fisher)[^206^](#_ENREF_206) and GT1a: to detect acute oropharyngeal palsy[^207^](#_ENREF_207)   Cytokines[^208^](#_ENREF_208):   - IFN-γ, TNF-α[^208^](#_ENREF_208) - IL-17A and IL-22[^209^](#_ENREF_209) - IL-12, IL-12R1[^210^](#_ENREF_210): elevated in acute inflammatory demyelinating polyneuropathy (AIDP) - CCL2[^211^](#_ENREF_211)   Other candidate biomarkers:   - C-reactive protein (CRP)[^212^](#_ENREF_212) - Serum albumin[^213^](#_ENREF_213) |

**References**

1. Petri M, Orbai AM, Alarcon GS, Gordon C, Merrill JT, Fortin PR, Bruce IN, Isenberg D, Wallace DJ, Nived O, Sturfelt G, Ramsey-Goldman R, Bae SC, Hanly JG, Sanchez-Guerrero J, Clarke A, Aranow C, Manzi S, Urowitz M, Gladman D, Kalunian K, Costner M, Werth VP, Zoma A, Bernatsky S, Ruiz-Irastorza G, Khamashta MA, Jacobsen S, Buyon JP, Maddison P, Dooley MA, van Vollenhoven RF, Ginzler E, Stoll T, Peschken C, Jorizzo JL, Callen JP, Lim SS, Fessler BJ, Inanc M, Kamen DL, Rahman A, Steinsson K, Franks AG, Jr., Sigler L, Hameed S, Fang H, Pham N, Brey R, Weisman MH, McGwin G, Jr. and Magder LS. Derivation and validation of the Systemic Lupus International Collaborating Clinics classification criteria for systemic lupus erythematosus. *Arthritis and rheumatism*. 2012;64:2677-86.

2. Nozawa K, Doe K, Uomori K, Sekigawa I, Takasaki Y, Yamaji K and Tamura N. Antiribonuclease H2 antibodies are an immune biomarker for systemic lupus erythematosus. *Autoimmunity*. 2017:1-6.

3. Serada S, Fujimoto M, Takahashi T, He P, Hayashi A, Tanaka T, Hagihara K, Yamadori T, Mochizuki M, Norioka N, Norioka S, Kawase I and Naka T. Proteomic analysis of autoantigens associated with systemic lupus erythematosus: Anti-aldolase A antibody as a potential marker of lupus nephritis. *Proteomics Clinical applications*. 2007;1:185-91.

4. Caster DJ, Korte EA, Merchant ML, Klein JB, Wilkey DW, Rovin BH, Birmingham DJ, Harley JB, Cobb BL, Namjou B, McLeish KR and Powell DW. Autoantibodies targeting glomerular annexin A2 identify patients with proliferative lupus nephritis. *Proteomics Clinical applications*. 2015;9:1012-20.

5. Sui W, Tang D, Zou G, Chen J, Ou M, Zhang Y and Dai Y. Differential proteomic analysis of renal tissue in lupus nephritis using iTRAQ reagent technology. *Rheumatol Int*. 2012;32:3537-43.

6. Crispín JC and Tsokos GC. IL-17 in Systemic Lupus Erythematosus. *Journal of Biomedicine and Biotechnology*. 2010;2010:4.

7. Wong CK, Ho CY, Li EK and Lam CWK. Elevation of proinflammatory cytokine (IL-18, IL-17, IL-12) and Th2 cytokine (IL-4) concentrations in patients with systemic lupus erythematosus. *Lupus*. 2000;9:589-593.

8. Nicolaou O, Kousios A, Hadjisavvas A, Lauwerys B, Sokratous K and Kyriacou K. Biomarkers of systemic lupus erythematosus identified using mass spectrometry‐based proteomics: a systematic review. *Journal of Cellular and Molecular Medicine*. 2017;21:993-1012.

9. Morgan PE, Sturgess AD, Hennessy A and Davies MJ. Serum protein oxidation and apolipoprotein CIII levels in people with systemic lupus erythematosus with and without nephritis. *Free radical research*. 2007;41:1301-12.

10. Yilmaz A, Sari RA, Gundogdu M, Kose N and Dag E. Trace elements and some extracellular antioxidant proteins levels in serum of patients with systemic lupus erythematosus. *Clinical rheumatology*. 2005;24:331-5.

11. Shen L, Suresh L, Lindemann M, Xuan J, Kowal P, Malyavantham K and Ambrus JL, Jr. Novel autoantibodies in Sjogren's syndrome. *Clinical immunology (Orlando, Fla)*. 2012;145:251-5.

12. Maria NI, Brkic Z, Waris M, van Helden-Meeuwsen CG, Heezen K, van de Merwe JP, van Daele PL, Dalm VA, Drexhage HA and Versnel MA. MxA as a clinically applicable biomarker for identifying systemic interferon type I in primary Sjogren's syndrome. *Annals of the rheumatic diseases*. 2014;73:1052-9.

13. Nishikawa A, Suzuki K, Kassai Y, Gotou Y, Takiguchi M, Miyazaki T, Yoshimoto K, Yasuoka H, Yamaoka K, Morita R, Yoshimura A and Takeuchi T. Identification of definitive serum biomarkers associated with disease activity in primary Sjögren’s syndrome. *Arthritis Research & Therapy*. 2016;18:106.

14. Ho KT and Reveille JD. The clinical relevance of autoantibodies in scleroderma. *Arthritis research & therapy*. 2003;5:80-93.

15. Steen VD. Autoantibodies in systemic sclerosis. *Seminars in arthritis and rheumatism*. 2005;35:35-42.

16. Koenig M, Dieude M and Senecal JL. Predictive value of antinuclear autoantibodies: the lessons of the systemic sclerosis autoantibodies. *Autoimmunity reviews*. 2008;7:588-93.

17. Degiannis D, Seibold JR, Czarnecki M, Raskova J and Raska K, Jr. Soluble interleukin-2 receptors in patients with systemic sclerosis. Clinical and laboratory correlations. *Arthritis and rheumatism*. 1990;33:375-80.

18. Steen VD, Engel EE, Charley MR and Medsger TA, Jr. Soluble serum interleukin 2 receptors in patients with systemic sclerosis. *The Journal of rheumatology*. 1996;23:646-9.

19. van Bon L, Affandi AJ, Broen J, Christmann RB, Marijnissen RJ, Stawski L, Farina GA, Stifano G, Mathes AL, Cossu M, York M, Collins C, Wenink M, Huijbens R, Hesselstrand R, Saxne T, DiMarzio M, Wuttge D, Agarwal SK, Reveille JD, Assassi S, Mayes M, Deng Y, Drenth JP, de Graaf J, den Heijer M, Kallenberg CG, Bijl M, Loof A, van den Berg WB, Joosten LA, Smith V, de Keyser F, Scorza R, Lunardi C, van Riel PL, Vonk M, van Heerde W, Meller S, Homey B, Beretta L, Roest M, Trojanowska M, Lafyatis R and Radstake TR. Proteome-wide analysis and CXCL4 as a biomarker in systemic sclerosis. *The New England journal of medicine*. 2014;370:433-43.

20. Castro SV and Jimenez SA. Biomarkers in systemic sclerosis. *Biomarkers in medicine*. 2010;4:133-147.

21. Hasegawa M. Biomarkers in systemic sclerosis: Their potential to predict clinical courses. *The Journal of dermatology*. 2016;43:29-38.

22. Soderstrom C, Berstein G, Zhang W, Valdez H, Fitz L, Kuhn M and Fraser S. Ultra-Sensitive Measurement of IL-17A and IL-17F in Psoriasis Patient Serum and Skin. *The AAPS journal*. 2017;19:1218-1222.

23. Arican O, Aral M, Sasmaz S and Ciragil P. Serum Levels of TNF-α, IFN-γ, IL-6, IL-8. *Mediators of Inflammation*. 2005;2005:273-279.

24. Meephansan J, Ruchusatsawat K, Sindhupak W, Thorner PS and Wongpiyabovorn J. Effect of methotrexate on serum levels of IL-22 in patients with psoriasis. *European journal of dermatology : EJD*. 2011;21:501-4.

25. Reindl J, Pesek J, Kruger T, Wendler S, Nemitz S, Muckova P, Buchler R, Opitz S, Krieg N, Norgauer J and Rhode H. Proteomic biomarkers for psoriasis and psoriasis arthritis. *Journal of proteomics*. 2016;140:55-61.

26. Plavina T, Wakshull E, Hancock WS and Hincapie M. Combination of abundant protein depletion and multi-lectin affinity chromatography (M-LAC) for plasma protein biomarker discovery. *Journal of proteome research*. 2007;6:662-71.

27. Plavina T, Hincapie M, Wakshull E, Subramanyam M and Hancock WS. Increased plasma concentrations of cytoskeletal and Ca2+-binding proteins and their peptides in psoriasis patients. *Clinical chemistry*. 2008;54:1805-14.

28. Garbaraviciene J, Diehl S, Varwig D, Bylaite M, Ackermann H, Ludwig RJ and Boehncke WH. Platelet P-selectin reflects a state of cutaneous inflammation: possible application to monitor treatment efficacy in psoriasis. *Experimental dermatology*. 2010;19:736-41.

29. Rocha-Pereira P, Santos-Silva A, Rebelo I, Figueiredo A, Quintanilha A and Teixeira F. The inflammatory response in mild and in severe psoriasis. *The British journal of dermatology*. 2004;150:917-28.

30. Boehncke S, Thaci D, Beschmann H, Ludwig RJ, Ackermann H, Badenhoop K and Boehncke WH. Psoriasis patients show signs of insulin resistance. *The British journal of dermatology*. 2007;157:1249-51.

31. Raghu G, Collard HR, Egan JJ, Martinez FJ, Behr J, Brown KK, Colby TV, Cordier JF, Flaherty KR, Lasky JA, Lynch DA, Ryu JH, Swigris JJ, Wells AU, Ancochea J, Bouros D, Carvalho C, Costabel U, Ebina M, Hansell DM, Johkoh T, Kim DS, King TE, Jr., Kondoh Y, Myers J, Muller NL, Nicholson AG, Richeldi L, Selman M, Dudden RF, Griss BS, Protzko SL and Schunemann HJ. An official ATS/ERS/JRS/ALAT statement: idiopathic pulmonary fibrosis: evidence-based guidelines for diagnosis and management. *Am J Respir Crit Care Med*. 2011;183:788-824.

32. Ziegenhagen MW, Zabel P, Zissel G, Schlaak M and Muller-Quernheim J. Serum level of interleukin 8 is elevated in idiopathic pulmonary fibrosis and indicates disease activity. *Am J Respir Crit Care Med*. 1998;157:762-8.

33. Tsoutsou PG, Gourgoulianis KI, Petinaki E, Germenis A, Tsoutsou AG, Mpaka M, Efremidou S and Molyvdas PA. Cytokine levels in the sera of patients with idiopathic pulmonary fibrosis. *Respiratory medicine*. 2006;100:938-45.

34. Ohshimo S, Ishikawa N, Horimasu Y, Hattori N, Hirohashi N, Tanigawa K, Kohno N, Bonella F, Guzman J and Costabel U. Baseline KL-6 predicts increased risk for acute exacerbation of idiopathic pulmonary fibrosis. *Respiratory medicine*. 2014;108:1031-9.

35. Ohshimo S, Yokoyama A, Hattori N, Ishikawa N, Hirasawa Y and Kohno N. KL-6, a human MUC1 mucin, promotes proliferation and survival of lung fibroblasts. *Biochemical and biophysical research communications*. 2005;338:1845-52.

36. Greene KE, King TE, Kuroki Y, Bucher-Bartelson B, Hunninghake GW, Newman LS, Nagae H and Mason RJ. Serum surfactant proteins‐A and ‐D as biomarkers in idiopathic pulmonary fibrosis. *European Respiratory Journal*. 2002;19:439.

37. Wang K, Ju Q, Cao J, Tang W and Zhang J. Impact of serum SP-A and SP-D levels on comparison and prognosis of idiopathic pulmonary fibrosis: A systematic review and meta-analysis. *Medicine (Baltimore)*. 2017;96:e7083.

38. Vij R and Noth I. Peripheral blood biomarkers in idiopathic pulmonary fibrosis. *Translational research : the journal of laboratory and clinical medicine*. 2012;159:218-27.

39. Dancer RC, Wood AM and Thickett DR. Metalloproteinases in idiopathic pulmonary fibrosis. *Eur Respir J*. 2011;38:1461-7.

40. Rosas IO, Richards TJ, Konishi K, Zhang Y, Gibson K, Lokshin AE, Lindell KO, Cisneros J, Macdonald SD, Pardo A, Sciurba F, Dauber J, Selman M, Gochuico BR and Kaminski N. MMP1 and MMP7 as potential peripheral blood biomarkers in idiopathic pulmonary fibrosis. *PLoS medicine*. 2008;5:e93.

41. Guiot J, Moermans C, Henket M, Corhay JL and Louis R. Blood Biomarkers in Idiopathic Pulmonary Fibrosis. *Lung*. 2017;195:273-280.

42. Miyakis S, Lockshin MD, Atsumi T, Branch DW, Brey RL, Cervera R, Derksen RH, PG DEG, Koike T, Meroni PL, Reber G, Shoenfeld Y, Tincani A, Vlachoyiannopoulos PG and Krilis SA. International consensus statement on an update of the classification criteria for definite antiphospholipid syndrome (APS). *Journal of thrombosis and haemostasis : JTH*. 2006;4:295-306.

43. Swadzba J, Iwaniec T, Pulka M, Laat BD, Groot PGD and Musial J. Lupus anticoagulant: performance of the tests as recommended by the latest ISTH guidelines. *Journal of Thrombosis and Haemostasis*. 2011;9:1776-1783.

44. Nash MJ, Camilleri RS, Kunka S, Mackie IJ, Machin SJ and Cohen H. The anticardiolipin assay is required for sensitive screening for antiphospholipid antibodies. *Journal of Thrombosis and Haemostasis*. 2004;2:1077-1081.

45. Reber G, Tincani A, Sanmarco M, Moerloose PD and Boffa MC. Proposals for the measurement of anti‐β2‐glycoprotein I antibodies. Standardization Group of the European Forum on Antiphospholipid Antibodies. *Journal of Thrombosis and Haemostasis*. 2004;2:1860-1862.

46. Hoxha A, Mattia E, Tonello M, Grava C, Pengo V and Ruffatti A. Antiphosphatidylserine/prothrombin antibodies as biomarkers to identify severe primary antiphospholipid syndrome. *Clinical chemistry and laboratory medicine*. 2017;55:890-898.

47. Lambert MP and Gernsheimer TB. Clinical updates in adult immune thrombocytopenia. *Blood*. 2017;129:2829-2835.

48. Hou M, Stockelberg D, Kutti J and Wadenvik H. Antibodies against platelet GPIb/IX, GPIIb/IIIa, and other platelet antigens in chronic idiopathic thrombocytopenic purpura. *European journal of haematology*. 1995;55:307-14.

49. McMillan R. Antiplatelet antibodies in chronic immune thrombocytopenia and their role in platelet destruction and defective platelet production. *Hematology/oncology clinics of North America*. 2009;23:1163-75.

50. Zhu XJ, Shi Y, Peng J, Guo CS, Shan NN, Qin P, Ji XB and Hou M. The effects of BAFF and BAFF-R-Fc fusion protein in immune thrombocytopenia. *Blood*. 2009;114:5362-7.

51. Gu D, Ge J, Du W, Xue F, Chen Z, Zhao H, Zhou Z, Xu J, Liu P, Zhao Q, Zhang L and Yang R. Raised expression of APRIL in Chinese patients with immune thrombocytopenia and its clinical implications. *Autoimmunity*. 2009;42:692-8.

52. Gu D, Chen Z, Zhao H, Du W, Xue F, Ge J, Sui T, Wu H, Liu B, Lu S, Zhang L and Yang R. Th1 (CXCL10) and Th2 (CCL2) chemokine expression in patients with immune thrombocytopenia. *Human immunology*. 2010;71:586-91.

53. Jernas M, Hou Y, Stromberg Celind F, Shao L, Wang Q, Ju X, Mellgren K, Wadenvik H, Hou M and Olsson B. Altered cytokine levels in pediatric ITP. *Platelets*. 2015;26:589-92.

54. DeGroot LJ. Diagnosis and Treatment of Grave's Disease. *De Groot LJ, Chrousos G, Dungan K, et al, editors Endotext [Internet]*. 2000.

55. Hayter SM and Cook MC. Updated assessment of the prevalence, spectrum and case definition of autoimmune disease. *Autoimmunity Reviews*. 2012;11:754-765.

56. Fröhlich E and Wahl R. Thyroid Autoimmunity: Role of Anti-thyroid Antibodies in Thyroid and Extra-Thyroidal Diseases. *Frontiers in Immunology*. 2017;8:521.

57. Martin CS, Sirbu AE, Betivoiu MA, Florea S, Barbu CG and Fica SV. Serum immunoglobulin G4 levels and Graves' disease phenotype. *Endocrine*. 2017;55:478-484.

58. Lin JD, Wang YH, Fang WF, Hsiao CJ, Chagnaadorj A, Lin YF, Tang KT and Cheng CW. Serum BAFF and thyroid autoantibodies in autoimmune thyroid disease. *Clinica chimica acta; international journal of clinical chemistry*. 2016;462:96-102.

59. Li X, Qi Y, Ma X, Huang F, Guo H, Jiang X, Hong J, Lin D, Cui B, Ning G, Xu L and Wang S. Chemokine (C-C motif) ligand 20, a potential biomarker for Graves' disease, is regulated by osteopontin. *PloS one*. 2013;8:e64277.

60. Song RH, Qin Q, Wang X, Yan N, Meng S, Shi XH, He ST and Zhang JA. Differential cytokine expression detected by protein microarray screening in peripheral blood of patients with refractory Graves' disease. *Clinical endocrinology*. 2016;84:402-7.

61. Hiratsuka I, Itoh M, Yamada H, Yamamoto K, Tomatsu E, Makino M, Hashimoto S and Suzuki A. Simultaneous measurement of serum chemokines in autoimmune thyroid diseases: possible role of IP-10 in the inflammatory response. *Endocrine journal*. 2015;62:1059-66.

62. Sun Z, Yi L, Tao H, Huang J, Jin Z, Xiao Y, Feng C and Sun J. Enhancement of soluble CD28 levels in the serum of Graves' disease. *Central-European journal of immunology*. 2014;39:216-22.

63. Celik HT, Abusoglu S, Burnik SF, Sezer S, Serdar MA, Ercan M, Uguz N, Avcikucuk M, Ceylan B and Yildirimkaya M. Increased serum interleukin-33 levels in patients with Graves' disease. *Endocrine regulations*. 2013;47:57-64.

64. Tilly N, Schneider JG, Leidig-Bruckner G, Sommer U and Kasperk C. Endothelin-1 levels in patients with disorders of the thyroid gland. *Experimental and clinical endocrinology & diabetes : official journal, German Society of Endocrinology [and] German Diabetes Association*. 2003;111:80-4.

65. Caturegli P, De Remigis A and Rose NR. Hashimoto thyroiditis: clinical and diagnostic criteria. *Autoimmunity reviews*. 2014;13:391-7.

66. Garber JR, Cobin RH, Gharib H, Hennessey JV, Klein I, Mechanick JI, Pessah-Pollack R, Singer PA and Woeber KA. Clinical practice guidelines for hypothyroidism in adults: cosponsored by the American Association of Clinical Endocrinologists and the American Thyroid Association. *Thyroid : official journal of the American Thyroid Association*. 2012;22:1200-35.

67. Kawashima ST, Tagami T, Nakao K, Nanba K, Tamanaha T, Usui T, Naruse M, Minamiguchi S, Mori Y, Tsuji J, Tanaka I and Shimatsu A. Serum levels of IgG and IgG4 in Hashimoto thyroiditis. *Endocrine*. 2014;45:236-43.

68. Yilmaz H, Cakmak M, Ceydilek B, Demir C and Aktas A. Role of interlekin-35 as a biomarker in patients with newly diagnosed Hashimoto's thyroiditis. *Endocrine regulations*. 2016;50:55-61.

69. Ignjatovic VD, Matovic MD, Vukomanovic VR, Jankovic SM and Dzodic RR. Is there a link between Hashimoto's thyroiditis and primary hyperparathyroidism? A study of serum parathormone and anti-TPO antibodies in 2267 patients. *Hellenic journal of nuclear medicine*. 2013;16:86-90.

70. Report of the Expert Committee on the Diagnosis and Classification of Diabetes Mellitus. *Diabetes Care*. 1997;20:1183-97.

71. Genuth S, Alberti KG, Bennett P, Buse J, Defronzo R, Kahn R, Kitzmiller J, Knowler WC, Lebovitz H, Lernmark A, Nathan D, Palmer J, Rizza R, Saudek C, Shaw J, Steffes M, Stern M, Tuomilehto J and Zimmet P. Follow-up report on the diagnosis of diabetes mellitus. *Diabetes Care*. 2003;26:3160-7.

72. American Diabetes A. Diagnosis and Classification of Diabetes Mellitus. *Diabetes Care*. 2010;33:S62-S69.

73. Taplin CE and Barker JM. Autoantibodies in type 1 diabetes. *Autoimmunity*. 2008;41:11-8.

74. Araujo DB, Skarstrand H, Barone B, Dantas JR, Kupfer R, Zajdenverg L, Milech A, Vaziri-Sani F, Oliveira JE and Rodacki M. Zinc transporter 8 autoantibodies in patients with type 1 diabetes from a multiethnic population and their first degree relatives. *Arquivos brasileiros de endocrinologia e metabologia*. 2014;58:737-43.

75. Yu L, Boulware DC, Beam CA, Hutton JC, Wenzlau JM, Greenbaum CJ, Bingley PJ, Krischer JP, Sosenko JM, Skyler JS, Eisenbarth GS and Mahon JL. Zinc transporter-8 autoantibodies improve prediction of type 1 diabetes in relatives positive for the standard biochemical autoantibodies. *Diabetes Care*. 2012;35:1213-8.

76. Park SG, Park HS, Jeong IK, Cho YM, Lee HK, Kang YS, Kim S and Park KS. Autoantibodies against aminoacyl-tRNA synthetase: novel diagnostic marker for type 1 diabetes mellitus. *Biomarkers : biochemical indicators of exposure, response, and susceptibility to chemicals*. 2010;15:358-66.

77. Takahashi K, Ohara M, Sasai T, Homma H, Nagasawa K, Takahashi T, Yamashina M, Ishii M, Fujiwara F, Kajiwara T, Taneichi H, Takebe N and Satoh J. Serum CXCL1 concentrations are elevated in type 1 diabetes mellitus, possibly reflecting activity of anti-islet autoimmune activity. *Diabetes/metabolism research and reviews*. 2011;27:830-3.

78. Jin Y and She J-X. Novel Biomarkers in Type 1 Diabetes. *The Review of Diabetic Studies : RDS*. 2012;9:224-235.

79. Purohit T and Cappell MS. Primary biliary cirrhosis: Pathophysiology, clinical presentation and therapy. *World Journal of Hepatology*. 2015;7:926-941.

80. Bowlus CL and Gershwin ME. The diagnosis of primary biliary cirrhosis. *Autoimmunity reviews*. 2014;13:441-4.

81. Yamagiwa S, Kamimura H, Takamura M and Aoyagi Y. Autoantibodies in primary biliary cirrhosis: recent progress in research on the pathogenetic and clinical significance. *World journal of gastroenterology*. 2014;20:2606-12.

82. Agmon-Levin N, Shapira Y, Selmi C, Barzilai O, Ram M, Szyper-Kravitz M, Sella S, Katz B-sP, Youinou P, Renaudineau Y, Larida B, Invernizzi P, Gershwin ME and Shoenfeld Y. A comprehensive evaluation of serum autoantibodies in primary biliary cirrhosis. *Journal of Autoimmunity*. 2010;34:55-58.

83. Granito A, Yang WH, Muratori L, Lim MJ, Nakajima A, Ferri S, Pappas G, Quarneti C, Bianchi FB, Bloch DB and Muratori P. PML nuclear body component Sp140 is a novel autoantigen in primary biliary cirrhosis. *The American journal of gastroenterology*. 2010;105:125-31.

84. Yamano T, Higashi T, Nouso K, Nakatsukasa H, Kariyama K, Yumoto E, Kobayashi Y, Yamamoto K, Iwagaki H, Yagi T, Tanimoto T, Kurimoto M, Tanaka N and Tsuji T. Serum interferon-gamma-inducing factor/IL-18 levels in primary biliary cirrhosis. *Clinical and experimental immunology*. 2000;122:227-31.

85. Neuman M, Angulo P, Malkiewicz I, Jorgensen R, Shear N, Dickson ER, Haber J, Katz G and Lindor K. Tumor necrosis factor-alpha and transforming growth factor-beta reflect severity of liver damage in primary biliary cirrhosis. *Journal of gastroenterology and hepatology*. 2002;17:196-202.

86. Krams SM, Cao S, Hayashi M, Villanueva JC and Martinez OM. Elevations in IFN-gamma, IL-5, and IL-10 in patients with the autoimmune disease primary biliary cirrhosis: association with autoantibodies and soluble CD30. *Clinical immunology and immunopathology*. 1996;80:311-20.

87. Alvarez F, Berg PA, Bianchi FB, Bianchi L, Burroughs AK, Cancado EL, Chapman RW, Cooksley WG, Czaja AJ, Desmet VJ, Donaldson PT, Eddleston AL, Fainboim L, Heathcote J, Homberg JC, Hoofnagle JH, Kakumu S, Krawitt EL, Mackay IR, MacSween RN, Maddrey WC, Manns MP, McFarlane IG, Meyer zum Buschenfelde KH, Zeniya M and et al. International Autoimmune Hepatitis Group Report: review of criteria for diagnosis of autoimmune hepatitis. *Journal of hepatology*. 1999;31:929-38.

88. Zachou K, Rigopoulou E and Dalekos GN. Autoantibodies and autoantigens in autoimmune hepatitis: important tools in clinical practice and to study pathogenesis of the disease. *Journal of Autoimmune Diseases*. 2004;1:2-2.

89. Oo YH, Hubscher SG and Adams DH. Autoimmune hepatitis: new paradigms in the pathogenesis, diagnosis, and management. *Hepatology International*. 2010;4:475-493.

90. Granito A, Muratori L, Muratori P, Pappas G, Guidi M, Cassani F, Volta U, Ferri A, Lenzi M and Bianchi FB. Antibodies to filamentous actin (F-actin) in type 1 autoimmune hepatitis. *Journal of clinical pathology*. 2006;59:280-4.

91. Manns MP, Czaja AJ, Gorham JD, Krawitt EL, Mieli-Vergani G, Vergani D and Vierling JM. Diagnosis and management of autoimmune hepatitis. *Hepatology (Baltimore, Md)*. 2010;51:2193-213.

92. Czaja AJ, Shums Z and Norman GL. Nonstandard antibodies as prognostic markers in autoimmune hepatitis. *Autoimmunity*. 2004;37:195-201.

93. Roozendaal C and Kallenberg CG. Anti-neutrophil cytoplasm autoantibodies (ANCA) in autoimmune liver diseases. *Hepato-gastroenterology*. 1999;46:3034-40.

94. Li H, Li G, Zhao X, Wu Y, Ma W, Liu Y, Gong F and Liang S. Complementary serum proteomic analysis of autoimmune hepatitis in mice and patients. *Journal of Translational Medicine*. 2013;11:146-146.

95. Durazzo M, Niro G, Premoli A, Morello E, Rizzotto ER, Gambino R, Bo S, Musso G, Cassader M, Pagano G and Floreani A. Type 1 autoimmune hepatitis and adipokines: new markers for activity and disease progression? *Journal of gastroenterology*. 2009;44:476-82.

96. Feuerstein JD and Cheifetz AS. Ulcerative colitis: epidemiology, diagnosis, and management. *Mayo Clinic proceedings*. 2014;89:1553-63.

97. Lobaton T, Bessissow T, De Hertogh G, Lemmens B, Maedler C, Van Assche G, Vermeire S, Bisschops R, Rutgeerts P, Bitton A, Afif W, Marcus V and Ferrante M. The Modified Mayo Endoscopic Score (MMES): A New Index for the Assessment of Extension and Severity of Endoscopic Activity in Ulcerative Colitis Patients. *Journal of Crohn's & colitis*. 2015;9:846-52.

98. Travis SP, Schnell D, Krzeski P, Abreu MT, Altman DG, Colombel JF, Feagan BG, Hanauer SB, Lemann M, Lichtenstein GR, Marteau PR, Reinisch W, Sands BE, Yacyshyn BR, Bernhardt CA, Mary JY and Sandborn WJ. Developing an instrument to assess the endoscopic severity of ulcerative colitis: the Ulcerative Colitis Endoscopic Index of Severity (UCEIS). *Gut*. 2012;61:535-42.

99. Joossens S, Reinisch W, Vermeire S, Sendid B, Poulain D, Peeters M, Geboes K, Bossuyt X, Vandewalle P, Oberhuber G, Vogelsang H, Rutgeerts P and Colombel JF. The value of serologic markers in indeterminate colitis: a prospective follow-up study. *Gastroenterology*. 2002;122:1242-7.

100. Bernstein CN, El-Gabalawy H, Sargent M, Landers C, Rawsthorne P, Elias B and Targan SR. Assessing inflammatory bowel disease-associated antibodies in Caucasian and First Nations cohorts. *Canadian journal of gastroenterology = Journal canadien de gastroenterologie*. 2011;25:269-73.

101. Cioffi M, Riegler G, Vietri MT, Pilla P, Caserta L, Carratu R, Sica V and Molinari AM. Serum p53 antibodies in patients affected with ulcerative colitis. *Inflammatory bowel diseases*. 2004;10:606-11.

102. Chakraborty S. IL23 as a novel serum based biomarker in ulcerative colitis- an editorial. *Gastroenterology and hepatology from bed to bench*. 2012;5:1-2.

103. Rodriguez-Peralvarez ML, Garcia-Sanchez V, Villar-Pastor CM, Gonzalez R, Iglesias-Flores E, Muntane J and Gomez-Camacho F. Role of serum cytokine profile in ulcerative colitis assessment. *Inflammatory bowel diseases*. 2012;18:1864-71.

104. Diaz-Jimenez D, Nunez LE, Beltran CJ, Candia E, Suazo C, Alvarez-Lobos M, Gonzalez MJ, Hermoso MA and Quera R. Soluble ST2: a new and promising activity marker in ulcerative colitis. *World journal of gastroenterology*. 2011;17:2181-90.

105. Serada S, Fujimoto M, Terabe F, Iijima H, Shinzaki S, Matsuzaki S, Ohkawara T, Nezu R, Nakajima S, Kobayashi T, Plevy SE, Takehara T and Naka T. Serum leucine-rich alpha-2 glycoprotein is a disease activity biomarker in ulcerative colitis. *Inflammatory bowel diseases*. 2012;18:2169-79.

106. Soendergaard C, Nielsen OH, Seidelin JB, Kvist PH and Bjerrum JT. Alpha-1 antitrypsin and granulocyte colony-stimulating factor as serum biomarkers of disease severity in ulcerative colitis. *Inflammatory bowel diseases*. 2015;21:1077-88.

107. Chouhan S, Gahlot S, Pokharna RK, Mathur KC, Saini K and Pal M. Severity and extent of ulcerative colitis: role of C-reactive protein. *Indian journal of gastroenterology : official journal of the Indian Society of Gastroenterology*. 2006;25:46-7.

108. Travis SP, Farrant JM, Ricketts C, Nolan DJ, Mortensen NM, Kettlewell MG and Jewell DP. Predicting outcome in severe ulcerative colitis. *Gut*. 1996;38:905-10.

109. Srivastava S, Kedia S, Kumar S, Pratap Mouli V, Dhingra R, Sachdev V, Tiwari V, Kurrey L, Pradhan R and Ahuja V. Serum human trefoil factor 3 is a biomarker for mucosal healing in ulcerative colitis patients with minimal disease activity. *Journal of Crohn's & colitis*. 2015;9:575-9.

110. Best WR, Becktel JM and Singleton JW. Rederived values of the eight coefficients of the Crohn's Disease Activity Index (CDAI). *Gastroenterology*. 1979;77:843-6.

111. Best WR, Becktel JM, Singleton JW and Kern F, Jr. Development of a Crohn's disease activity index. National Cooperative Crohn's Disease Study. *Gastroenterology*. 1976;70:439-44.

112. Gomollón F, Dignass A, Annese V, Tilg H, Van Assche G, Lindsay JO, Peyrin-Biroulet L, Cullen GJ, Daperno M, Kucharzik T, Rieder F, Almer S, Armuzzi A, Harbord M, Langhorst J, Sans M, Chowers Y, Fiorino G, Juillerat P, Mantzaris GJ, Rizzello F, Vavricka S and Gionchetti P. 3rd European Evidence-based Consensus on the Diagnosis and Management of Crohn’s Disease 2016: Part 1: Diagnosis and Medical Management. *Journal of Crohn's and Colitis*. 2017;11:3-25.

113. Wen Z and Fiocchi C. Inflammatory bowel disease: autoimmune or immune-mediated pathogenesis? *Clinical & developmental immunology*. 2004;11:195-204.

114. Dotan N, Altstock RT, Schwarz M and Dukler A. Anti-glycan antibodies as biomarkers for diagnosis and prognosis. *Lupus*. 2006;15:442-50.

115. Vandewalle-El Khoury P, Colombel JF, Joossens S, Standaert-Vitse A, Collot M, Halfvarson J, Ayadi A, Landers CJ, Vermeire S, Rutgeerts P, Targan SR, Chamaillard M, Mallet JM, Sendid B and Poulain D. Detection of antisynthetic mannoside antibodies (ASigmaMA) reveals heterogeneity in the ASCA response of Crohn's disease patients and contributes to differential diagnosis, stratification, and prediction. *The American journal of gastroenterology*. 2008;103:949-57.

116. Beaven SW and Abreu MT. Biomarkers in inflammatory bowel disease. *Current opinion in gastroenterology*. 2004;20:318-27.

117. Scoville EA, Allaman MM, Brown C, Motley A, Peyton SC, Horst SN, Williams CS, Adams DW, Beaulieu D, Schwartz DA, Wilson KT and Coburn LA. Utility of Serum Cytokine Analysis by Luminex-Based Multi-Analyte Testing in Crohn's Disease for Detecting Therapeutic Targets, Including TNF-&#x3b1; and IL-12P40. *Gastroenterology*. 152:S761.

118. Meuwis MA, Vernier-Massouille G, Grimaud JC, Bouhnik Y, Laharie D, Piver E, Seidel L, Colombel JF and Louis E. Serum calprotectin as a biomarker for Crohn's disease. *Journal of Crohn's & colitis*. 2013;7:e678-83.

119. Erzin Y, Uzun H, Karatas A and Celik AF. Serum YKL-40 as a marker of disease activity and stricture formation in patients with Crohn's disease. *Journal of gastroenterology and hepatology*. 2008;23:e357-62.

120. Rubio-Tapia A, Hill ID, Kelly CP, Calderwood AH and Murray JA. ACG clinical guidelines: diagnosis and management of celiac disease. *The American journal of gastroenterology*. 2013;108:656-76; quiz 677.

121. Ludvigsson JF, Bai JC, Biagi F, Card TR, Ciacci C, Ciclitira PJ, Green PHR, Hadjivassiliou M, Holdoway A, van Heel DA, Kaukinen K, Leffler DA, Leonard JN, Lundin KEA, McGough N, Davidson M, Murray JA, Swift GL, Walker MM, Zingone F and Sanders DS. Diagnosis and management of adult coeliac disease: guidelines from the British Society of Gastroenterology. *Gut*. 2014.

122. Kelly CP, Bai JC, Liu E and Leffler DA. Advances in diagnosis and management of celiac disease. *Gastroenterology*. 2015;148:1175-86.

123. Tonutti E, Visentini D, Bizzaro N, Caradonna M, Cerni L, Villalta D and Tozzoli R. The role of antitissue transglutaminase assay for the diagnosis and monitoring of coeliac disease: a French-Italian multicentre study. *Journal of clinical pathology*. 2003;56:389-93.

124. Volta U, Fabbri A, Parisi C, Piscaglia M, Caio G, Tovoli F and Fiorini E. Old and new serological tests for celiac disease screening. *Expert review of gastroenterology & hepatology*. 2010;4:31-5.

125. Collin P, Kaukinen K, Vogelsang H, Korponay-Szabo I, Sommer R, Schreier E, Volta U, Granito A, Veronesi L, Mascart F, Ocmant A, Ivarsson A, Lagerqvist C, Burgin-Wolff A, Hadziselimovic F, Furlano RI, Sidler MA, Mulder CJ, Goerres MS, Mearin ML, Ninaber MK, Gudmand-Hoyer E, Fabiani E, Catassi C, Tidlund H, Alainentalo L and Maki M. Antiendomysial and antihuman recombinant tissue transglutaminase antibodies in the diagnosis of coeliac disease: a biopsy-proven European multicentre study. *European journal of gastroenterology & hepatology*. 2005;17:85-91.

126. Hill ID. What are the sensitivity and specificity of serologic tests for celiac disease? Do sensitivity and specificity vary in different populations? *Gastroenterology*. 2005;128:S25-32.

127. Prince HE. Evaluation of the INOVA diagnostics enzyme-linked immunosorbent assay kits for measuring serum immunoglobulin G (IgG) and IgA to deamidated gliadin peptides. *Clinical and vaccine immunology : CVI*. 2006;13:150-1.

128. Manavalan JS, Hernandez L, Shah JG, Konikkara J, Naiyer AJ, Lee AR, Ciaccio E, Minaya MT, Green PH and Bhagat G. Serum cytokine elevations in celiac disease: association with disease presentation. *Human immunology*. 2010;71:50-7.

129. Adriaanse MP, Tack GJ, Passos VL, Damoiseaux JG, Schreurs MW, van Wijck K, Riedl RG, Masclee AA, Buurman WA, Mulder CJ and Vreugdenhil AC. Serum I-FABP as marker for enterocyte damage in coeliac disease and its relation to villous atrophy and circulating autoantibodies. *Alimentary pharmacology & therapeutics*. 2013;37:482-90.

130. Garrett S, Jenkinson T, Kennedy LG, Whitelock H, Gaisford P and Calin A. A new approach to defining disease status in ankylosing spondylitis: the Bath Ankylosing Spondylitis Disease Activity Index. *The Journal of rheumatology*. 1994;21:2286-91.

131. Calin A, Garrett S, Whitelock H, Kennedy LG, O'Hea J, Mallorie P and Jenkinson T. A new approach to defining functional ability in ankylosing spondylitis: the development of the Bath Ankylosing Spondylitis Functional Index. *The Journal of rheumatology*. 1994;21:2281-5.

132. van der Heijde D, Lie E, Kvien TK, Sieper J, Van den Bosch F, Listing J, Braun J and Landewe R. ASDAS, a highly discriminatory ASAS-endorsed disease activity score in patients with ankylosing spondylitis. *Ann Rheum Dis*. 2009;68:1811-8.

133. Baerlecken NT, Nothdorft S, Stummvoll GH, Sieper J, Rudwaleit M, Reuter S, Matthias T, Schmidt RE and Witte T. Autoantibodies against CD74 in spondyloarthritis. *Annals of the rheumatic diseases*. 2014;73:1211-1214.

134. Baraliakos X, Baerlecken N, Witte T, Heldmann F and Braun J. High prevalence of anti-CD74 antibodies specific for the HLA class II-associated invariant chain peptide (CLIP) in patients with axial spondyloarthritis. *Annals of the rheumatic diseases*. 2014;73:1079-1082.

135. Wright C, Sibani S, Trudgian D, Fischer R, Kessler B, LaBaer J and Bowness P. Detection of multiple autoantibodies in patients with ankylosing spondylitis using nucleic acid programmable protein arrays. *Molecular & cellular proteomics : MCP*. 2012;11:M9.00384.

136. Kim Y-G, Sohn DH, Zhao X, Sokolove J, Lindstrom TM, Yoo B, Lee C-K, Reveille JD, Taurog JD and Robinson WH. Role of Protein Phosphatase Magnesium-Dependent 1A and Anti–Protein Phosphatase Magnesium-Dependent 1A Autoantibodies in Ankylosing Spondylitis. *Arthritis & Rheumatology*. 2014;66:2793-2803.

137. Garg N, Krishan P and Syngle A. Rosuvastatin improves endothelial dysfunction in ankylosing spondylitis. *Clinical rheumatology*. 2015;34:1065-71.

138. Visvanathan S, Wagner C, Marini JC, Baker D, Gathany T, Han J, van der Heijde D and Braun J. Inflammatory biomarkers, disease activity and spinal disease measures in patients with ankylosing spondylitis after treatment with infliximab. *Annals of the Rheumatic Diseases*. 2008;67:511-517.

139. He D, Zhu Q, Zhou Q, Qi Q, Sun H, Zachariah LM, Wang G, Reveille JD, Guan Y and Zhou X. Correlation of serum MMP3 and other biomarkers with clinical outcomes in patients with ankylosing spondylitis: a pilot study. *Clinical rheumatology*. 2017;36:1819-1826.

140. Baeten D, Baraliakos X, Braun J, Sieper J, Emery P, van der Heijde D, McInnes I, van Laar JM, Landewé R, Wordsworth P, Wollenhaupt J, Kellner H, Paramarta J, Wei J, Brachat A, Bek S, Laurent D, Li Y, Wang YA, Bertolino AP, Gsteiger S, Wright AM and Hueber W. Anti-interleukin-17A monoclonal antibody secukinumab in treatment of ankylosing spondylitis: a randomised, double-blind, placebo-controlled trial. *The Lancet*. 382:1705-1713.

141. Braun J, Deodhar A, Dijkmans B, Geusens P, Sieper J, Williamson P, Xu W, Visvanathan S, Baker D, Goldstein N and van der Heijde D. Efficacy and safety of infliximab in patients with ankylosing spondylitis over a two-year period. *Arthritis and rheumatism*. 2008;59:1270-8.

142. Arends S, Brouwer E, Efde M, van der Veer E, Bootsma H, Wink F and Spoorenberg A. Long-term drug survival and clinical effectiveness of etanercept treatment in patients with ankylosing spondylitis in daily clinical practice. *Clinical and experimental rheumatology*. 2017;35:61-68.

143. van der Heijde D, Schiff MH, Sieper J, Kivitz AJ, Wong RL, Kupper H, Dijkmans BA, Mease PJ and Davis JC, Jr. Adalimumab effectiveness for the treatment of ankylosing spondylitis is maintained for up to 2 years: long-term results from the ATLAS trial. *Annals of the rheumatic diseases*. 2009;68:922-9.

144. Deodhar A, Braun J, Inman RD, van der Heijde D, Zhou Y, Xu S, Han C and Hsu B. Golimumab administered subcutaneously every 4 weeks in ankylosing spondylitis: 5-year results of the GO-RAISE study. *Annals of the rheumatic diseases*. 2015;74:757-61.

145. Maksymowych WP, Poole AR, Hiebert L, Webb A, Ionescu M, Lobanok T, King L and Davis JC, Jr. Etanercept exerts beneficial effects on articular cartilage biomarkers of degradation and turnover in patients with ankylosing spondylitis. *The Journal of rheumatology*. 2005;32:1911-7.

146. Quaden DH, De Winter LM and Somers V. Detection of novel diagnostic antibodies in ankylosing spondylitis: An overview. *Autoimmunity reviews*. 2016;15:820-32.

147. Pathan E, Abraham S, Van Rossen E, Withrington R, Keat A, Charles PJ, Paterson E, Chowdhury M, McClinton C and Taylor PC. Efficacy and safety of apremilast, an oral phosphodiesterase 4 inhibitor, in ankylosing spondylitis. *Annals of the rheumatic diseases*. 2013;72:1475-80.

148. Prevoo ML, van 't Hof MA, Kuper HH, van Leeuwen MA, van de Putte LB and van Riel PL. Modified disease activity scores that include twenty-eight-joint counts. Development and validation in a prospective longitudinal study of patients with rheumatoid arthritis. *Arthritis and rheumatism*. 1995;38:44-8.

149. Aletaha D, Neogi T, Silman AJ, Funovits J, Felson DT, Bingham CO, 3rd, Birnbaum NS, Burmester GR, Bykerk VP, Cohen MD, Combe B, Costenbader KH, Dougados M, Emery P, Ferraccioli G, Hazes JM, Hobbs K, Huizinga TW, Kavanaugh A, Kay J, Kvien TK, Laing T, Mease P, Menard HA, Moreland LW, Naden RL, Pincus T, Smolen JS, Stanislawska-Biernat E, Symmons D, Tak PP, Upchurch KS, Vencovsky J, Wolfe F and Hawker G. 2010 rheumatoid arthritis classification criteria: an American College of Rheumatology/European League Against Rheumatism collaborative initiative. *Annals of the rheumatic diseases*. 2010;69:1580-8.

150. Wolfe F, Michaud K and Pincus T. A composite disease activity scale for clinical practice, observational studies, and clinical trials: the patient activity scale (PAS/PAS-II). *The Journal of rheumatology*. 2005;32:2410-5.

151. Pincus T, Swearingen CJ, Bergman M and Yazici Y. RAPID3 (Routine Assessment of Patient Index Data 3), a rheumatoid arthritis index without formal joint counts for routine care: proposed severity categories compared to disease activity score and clinical disease activity index categories. *The Journal of rheumatology*. 2008;35:2136-47.

152. Smolen JS, Breedveld FC, Schiff MH, Kalden JR, Emery P, Eberl G, van Riel PL and Tugwell P. A simplified disease activity index for rheumatoid arthritis for use in clinical practice. *Rheumatology (Oxford, England)*. 2003;42:244-57.

153. Anderson JK, Zimmerman L, Caplan L and Michaud K. Measures of rheumatoid arthritis disease activity: Patient (PtGA) and Provider (PrGA) Global Assessment of Disease Activity, Disease Activity Score (DAS) and Disease Activity Score with 28-Joint Counts (DAS28), Simplified Disease Activity Index (SDAI), Clinical Disease Activity Index (CDAI), Patient Activity Score (PAS) and Patient Activity Score-II (PASII), Routine Assessment of Patient Index Data (RAPID), Rheumatoid Arthritis Disease Activity Index (RADAI) and Rheumatoid Arthritis Disease Activity Index-5 (RADAI-5), Chronic Arthritis Systemic Index (CASI), Patient-Based Disease Activity Score With ESR (PDAS1) and Patient-Based Disease Activity Score without ESR (PDAS2), and Mean Overall Index for Rheumatoid Arthritis (MOI-RA). *Arthritis care & research*. 2011;63 Suppl 11:S14-36.

154. Silosi I, Boldeanu L, Biciusca V, Bogdan M, Avramescu C, Taisescu C, Padureanu V, Boldeanu MV, Dricu A and Silosi CA. Serum Biomarkers for Discrimination between Hepatitis C-Related Arthropathy and Early Rheumatoid Arthritis. *International journal of molecular sciences*. 2017;18.

155. Cordero OJ, Varela-Calvino R, Lopez-Gonzalez T, Grujic M, Juranic Z, Mourino C, Hernandez-Rodriguez I, Rodriguez-Lopez M, de la Iglesia BA and Pego-Reigosa JM. Anti-CD26 autoantibodies are involved in rheumatoid arthritis and show potential clinical interest. *Clinical biochemistry*. 2017.

156. Mjaavatten MD, van der Heijde D, Uhlig T, Haugen AJ, Nygaard H, Sidenvall G, Helgetveit K and Kvien TK. The likelihood of persistent arthritis increases with the level of anti-citrullinated peptide antibody and immunoglobulin M rheumatoid factor: a longitudinal study of 376 patients with very early undifferentiated arthritis. *Arthritis research & therapy*. 2010;12:R76.

157. Shi R, Chen M and Litifu B. Serum interleukin-6 and survivin levels predict clinical response to etanercept treatment in patients with established rheumatoid arthritis. *Modern rheumatology*. 2017:1-7.

158. Maksymowych WP, Naides SJ, Bykerk V, Siminovitch KA, van Schaardenburg D, Boers M, Landewe R, van der Heijde D, Tak PP, Genovese MC, Weinblatt ME, Keystone EC, Zhukov OS, Abolhosn RW, Popov JM, Britsemmer K, van Kuijk AW and Marotta A. Serum 14-3-3eta is a novel marker that complements current serological measurements to enhance detection of patients with rheumatoid arthritis. *The Journal of rheumatology*. 2014;41:2104-13.

159. Zheng X, Wu SL, Hincapie M and Hancock WS. Study of the human plasma proteome of rheumatoid arthritis. *Journal of chromatography A*. 2009;1216:3538-45.

160. Song R, Choi HM, Yang HI, Yoo MC, Park YB and Kim KS. Association between serum thymosin beta4 levels of rheumatoid arthritis patients and disease activity and response to therapy. *Clinical rheumatology*. 2012;31:1253-8.

161. Bae SC and Lee YH. Calprotectin levels in rheumatoid arthritis and their correlation with disease activity: a meta-analysis. *Postgraduate medicine*. 2017;129:531-537.

162. Chou CL, Wu MJ, Yu CL, Lu MC, Hsieh SC, Wu TH, Chou CT and Tsai CY. Anti-agalactosyl IgG antibody in ankylosing spondylitis and psoriatic arthritis. *Clinical rheumatology*. 2010;29:875-81.

163. Dalmady S, Kiss M, Kepiro L, Kovacs L, Sonkodi G, Kemeny L and Gyulai R. Higher levels of autoantibodies targeting mutated citrullinated vimentin in patients with psoriatic arthritis than in patients with psoriasis vulgaris. *Clinical & developmental immunology*. 2013;2013:474028.

164. Maejima H, Aki R, Watarai A, Shirai K, Hamada Y and Katsuoka K. Antibodies against cyclic citrullinated peptide in Japanese psoriatic arthritis patients. *The Journal of dermatology*. 2010;37:339-45.

165. Alenius GM, Eriksson C and Rantapaa Dahlqvist S. Interleukin-6 and soluble interleukin-2 receptor alpha-markers of inflammation in patients with psoriatic arthritis? *Clinical and experimental rheumatology*. 2009;27:120-3.

166. Chandran V, Cook RJ, Edwin J, Shen H, Pellett FJ, Shanmugarajah S, Rosen CF and Gladman DD. Soluble biomarkers differentiate patients with psoriatic arthritis from those with psoriasis without arthritis. *Rheumatology (Oxford, England)*. 2010;49:1399-405.

167. Cretu D, Gao L, Liang K, Soosaipillai A, Diamandis EP and Chandran V. Novel serum biomarkers differentiate psoriatic arthritis from psoriasis without psoriatic arthritis. *Arthritis care & research*. 2017.

168. Jensen P, Wiell C, Milting K, Poggenborg RP, Ostergaard M, Johansen JS and Skov L. Plasma YKL-40: a potential biomarker for psoriatic arthritis? *Journal of the European Academy of Dermatology and Venereology : JEADV*. 2013;27:815-9.

169. Dalbeth N, Pool B, Smith T, Callon KE, Lobo M, Taylor WJ, Jones PB, Cornish J and McQueen FM. Circulating mediators of bone remodeling in psoriatic arthritis: implications for disordered osteoclastogenesis and bone erosion. *Arthritis research & therapy*. 2010;12:R164.

170. Polman CH, Reingold SC, Banwell B, Clanet M, Cohen JA, Filippi M, Fujihara K, Havrdova E, Hutchinson M, Kappos L, Lublin FD, Montalban X, O'Connor P, Sandberg-Wollheim M, Thompson AJ, Waubant E, Weinshenker B and Wolinsky JS. Diagnostic criteria for multiple sclerosis: 2010 Revisions to the McDonald criteria. *Annals of Neurology*. 2011;69:292-302.

171. Kurtzke JF. Rating neurologic impairment in multiple sclerosis: an expanded disability status scale (EDSS). *Neurology*. 1983;33.

172. Schirmer L, Srivastava R and Hemmer B. To look for a needle in a haystack: the search for autoantibodies in multiple sclerosis. *Mult Scler*. 2014;20:271-9.

173. Brettschneider J, Jaskowski TD, Tumani H, Abdul S, Husebye D, Seraj H, Hill HR, Fire E, Spector L, Yarden J, Dotan N and Rose JW. Serum anti-GAGA4 IgM antibodies differentiate relapsing remitting and secondary progressive multiple sclerosis from primary progressive multiple sclerosis and other neurological diseases. *Journal of neuroimmunology*. 2009;217:95-101.

174. Mathey EK, Derfuss T, Storch MK, Williams KR, Hales K, Woolley DR, Al-Hayani A, Davies SN, Rasband MN, Olsson T, Moldenhauer A, Velhin S, Hohlfeld R, Meinl E and Linington C. Neurofascin as a novel target for autoantibody-mediated axonal injury. *The Journal of experimental medicine*. 2007;204:2363-72.

175. Srivastava R, Aslam M, Kalluri SR, Schirmer L, Buck D, Tackenberg B, Rothhammer V, Chan A, Gold R, Berthele A, Bennett JL, Korn T and Hemmer B. Potassium channel KIR4.1 as an immune target in multiple sclerosis. *The New England journal of medicine*. 2012;367:115-23.

176. Quintana FJ, Farez MF, Izquierdo G, Lucas M, Cohen IR and Weiner HL. Antigen microarrays identify CNS-produced autoantibodies in RRMS. *Neurology*. 2012;78:532-9.

177. Katsavos S and Anagnostouli M. Biomarkers in Multiple Sclerosis: An Up-to-Date Overview. *Multiple Sclerosis International*. 2013;2013:20.

178. Shajarian M, Alsahebfosoul F, Etemadifar M, Sedaghat N, Shahbazi M, Firouzabadi FP and Dezashibi HM. IL-23 plasma level measurement in relapsing remitting multiple sclerosis (RRMS) patients compared to healthy subjects. *Immunological investigations*. 2015;44:36-44.

179. Kallaur AP, Oliveira SR, Colado Simao AN, Delicato de Almeida ER, Kaminami Morimoto H, Lopes J, de Carvalho Jennings Pereira WL, Marques Andrade R, Muliterno Pelegrino L, Donizete Borelli S, Kaimen-Maciel DR and Reiche EM. Cytokine profile in relapsingremitting multiple sclerosis patients and the association between progression and activity of the disease. *Molecular medicine reports*. 2013;7:1010-20.

180. Martins TB, Rose JW, Jaskowski TD, Wilson AR, Husebye D, Seraj HS and Hill HR. Analysis of proinflammatory and anti-inflammatory cytokine serum concentrations in patients with multiple sclerosis by using a multiplexed immunoassay. *American journal of clinical pathology*. 2011;136:696-704.

181. Tejera-Alhambra M, Casrouge A, de Andres C, Seyfferth A, Ramos-Medina R, Alonso B, Vega J, Fernandez-Paredes L, Albert ML and Sanchez-Ramon S. Plasma biomarkers discriminate clinical forms of multiple sclerosis. *PLoS One*. 2015;10:e0128952.

182. Stenberg J, Elovsson M, Strehl R, Kilmare E, Hyllner J and Lindahl A. Sustained embryoid body formation and culture in a non-laborious three dimensional culture system for human embryonic stem cells. *Cytotechnology*. 2011;63:227-37.

183. Wang H, Wang K, Wang C, Zhong X, Qiu W and Hu X. Increased plasma levels of pentraxin 3 in patients with multiple sclerosis and neuromyelitis optica. *Mult Scler*. 2013;19:926-31.

184. Sussman J, Farrugia ME, Maddison P, Hill M, Leite MI and Hilton-Jones D. Myasthenia gravis: Association of British Neurologists’ management guidelines. *Practical Neurology*. 2015;15:199-206.

185. Gilhus NE, Skeie GO, Romi F, Lazaridis K, Zisimopoulou P and Tzartos S. Myasthenia gravis - autoantibody characteristics and their implications for therapy. *Nature reviews Neurology*. 2016;12:259-68.

186. Kaminski HJ, Kusner LL, Wolfe GI, Aban I, Minisman G, Conwit R and Cutter G. Biomarker development for myasthenia gravis. *Annals of the New York Academy of Sciences*. 2012;1275:101-6.

187. Gasperi C, Melms A, Schoser B, Zhang Y, Meltoranta J, Risson V, Schaeffer L, Schalke B and Kroger S. Anti-agrin autoantibodies in myasthenia gravis. *Neurology*. 2014;82:1976-83.

188. Zhang B, Shen C, Bealmear B, Ragheb S, Xiong WC, Lewis RA, Lisak RP and Mei L. Autoantibodies to agrin in myasthenia gravis patients. *PLoS One*. 2014;9:e91816.

189. Szczudlik P, Szyluk B, Lipowska M, Ryniewicz B, Kubiszewska J, Dutkiewicz M, Gilhus NE and Kostera-Pruszczyk A. Antititin antibody in early- and late-onset myasthenia gravis. *Acta Neurol Scand*. 2014;130:229-33.

190. Romi F, Suzuki S, Suzuki N, Petzold A, Plant GT and Gilhus NE. Anti-voltage-gated potassium channel Kv1.4 antibodies in myasthenia gravis. *J Neurol*. 2012;259:1312-6.

191. Skeie GO, Mygland A, Treves S, Gilhus NE, Aarli JA and Zorzato F. Ryanodine receptor antibodies in myasthenia gravis: epitope mapping and effect on calcium release in vitro. *Muscle & nerve*. 2003;27:81-83.

192. Zoltowska Katarzyna M, Belaya K, Leite M, Patrick W, Vincent A and Beeson D. Collagen Q--a potential target for autoantibodies in myasthenia gravis. *J Neurol Sci*. 2015;348:241-4.

193. Zisimopoulou P, Brenner T, Trakas N and Tzartos SJ. Serological diagnostics in myasthenia gravis based on novel assays and recently identified antigens. *Autoimmun Rev*. 2013;12:924-30.

194. Zhang DQ, Wang R, Li T, Li X, Qi Y, Wang J and Yang L. Remarkably increased resistin levels in anti-AChR antibody-positive myasthenia gravis. *Journal of neuroimmunology*. 2015;283:7-10.

195. Shiao YM, Lee CC, Hsu YH, Huang SF, Lin CY, Li LH, Fann CS, Tsai CY, Tsai SF and Chiu HC. Ectopic and high CXCL13 chemokine expression in myasthenia gravis with thymic lymphoid hyperplasia. *Journal of neuroimmunology*. 2010;221:101-6.

196. Molin CJ, Westerberg E and Punga AR. Profile of upregulated inflammatory proteins in sera of Myasthenia Gravis patients. *Sci Rep*. 2017;7:39716.

197. Asbury AK and Cornblath DR. Assessment of current diagnostic criteria for Guillain-Barre syndrome. *Ann Neurol*. 1990;27 Suppl:S21-4.

198. van den Berg B, Walgaard C, Drenthen J, Fokke C, Jacobs BC and van Doorn PA. Guillain-Barre syndrome: pathogenesis, diagnosis, treatment and prognosis. *Nature reviews Neurology*. 2014;10:469-82.

199. Fokke C, van den Berg B, Drenthen J, Walgaard C, van Doorn PA and Jacobs BC. Diagnosis of Guillain-Barre syndrome and validation of Brighton criteria. *Brain : a journal of neurology*. 2014;137:33-43.

200. Vanhoutte EK, Faber CG and Merkies IS. 196th ENMC international workshop: Outcome measures in inflammatory peripheral neuropathies 8-10 February 2013, Naarden, The Netherlands. *Neuromuscular disorders : NMD*. 2013;23:924-33.

201. van Nes SI, Vanhoutte EK, van Doorn PA, Hermans M, Bakkers M, Kuitwaard K, Faber CG and Merkies IS. Rasch-built Overall Disability Scale (R-ODS) for immune-mediated peripheral neuropathies. *Neurology*. 2011;76:337-45.

202. Kleyweg RP, van der Meche FG and Schmitz PI. Interobserver agreement in the assessment of muscle strength and functional abilities in Guillain-Barre syndrome. *Muscle & nerve*. 1991;14:1103-9.

203. Vanhoutte EK, Faber CG, van Nes SI, Jacobs BC, van Doorn PA, van Koningsveld R, Cornblath DR, van der Kooi AJ, Cats EA, van den Berg LH, Notermans NC, van der Pol WL, Hermans MC, van der Beek NA, Gorson KC, Eurelings M, Engelsman J, Boot H, Meijer RJ, Lauria G, Tennant A and Merkies IS. Modifying the Medical Research Council grading system through Rasch analyses. *Brain : a journal of neurology*. 2012;135:1639-49.

204. Gabriel CM, Gregson NA and Hughes RA. Anti-PMP22 antibodies in patients with inflammatory neuropathy. *Journal of neuroimmunology*. 2000;104:139-46.

205. Inglis HR, Csurhes PA and McCombe PA. Antibody responses to peptides of peripheral nerve myelin proteins P0 and P2 in patients with inflammatory demyelinating neuropathy. *Journal of Neurology, Neurosurgery, and Psychiatry*. 2007;78:419-422.

206. Chiba A, Kusunoki S, Shimizu T and Kanazawa I. Serum IgG antibody to ganglioside GQ1b is a possible marker of Miller Fisher syndrome. *Ann Neurol*. 1992;31:677-9.

207. O'Leary CP, Veitch J, Durward WF, Thomas AM, Rees JH and Willison HJ. Acute oropharyngeal palsy is associated with antibodies to GQ1b and GT1a gangliosides. *J Neurol Neurosurg Psychiatry*. 1996;61:649-51.

208. Wang Y, Sun S, Zhu J, Cui L and Zhang HL. Biomarkers of Guillain-Barre Syndrome: Some Recent Progress, More Still to Be Explored. *Mediators of inflammation*. 2015;2015:564098.

209. Li S, Jin T, Zhang HL, Yu H, Meng F, Concha Quezada H and Zhu J. Circulating Th17, Th22, and Th1 cells are elevated in the Guillain-Barre syndrome and downregulated by IVIg treatments. *Mediators of inflammation*. 2014;2014:740947.

210. Deng H, Yang X, Jin T, Wu J, Hu LS, Chang M, Sun XJ, Adem A, Winblad B and Zhu J. The role of IL-12 and TNF-alpha in AIDP and AMAN. *European journal of neurology*. 2008;15:1100-5.

211. Orlikowski D, Chazaud B, Plonquet A, Poron F, Sharshar T, Maison P, Raphael JC, Gherardi RK and Creange A. Monocyte chemoattractant protein 1 and chemokine receptor CCR2 productions in Guillain-Barre syndrome and experimental autoimmune neuritis. *Journal of neuroimmunology*. 2003;134:118-27.

212. Vaishnavi C, Kapoor P, Behura C, Singh SK and Prabhakar S. C-reactive protein in patients with Guillain Barre syndrome. *Indian journal of pathology & microbiology*. 2014;57:51-4.

213. Fokkink WR, Walgaard C, Kuitwaard K, Tio-Gillen AP, van Doorn PA and Jacobs BC. Association of albumin levels with outcome in intravenous immunoglobulin–treated guillain-barré syndrome. *JAMA Neurology*. 2017;74:189-196.
